# 6‑Chloropyridinyl Benzoate Fragments as Selective Anti-Melanoma Scaffolds Triggering ROS-Mediated Mitochondrial Apoptosis

**DOI:** 10.1021/acsomega.6c06714

**Published:** 2026-10-12

**Authors:** Gabriele La Monica, Ignazio Restivo, Alessia Bono, Federica Alamia, Dennis Tocco, Alessandro Attanzio, Antonino Lauria, Annamaria Martorana

**Affiliations:** † Dipartimento di Scienze e Tecnologie Biologiche Chimiche e Farmaceutiche “STEBICEF”, 18998 University of Palermo, 90123 Palermo, Italy; ‡ Fondazione Umberto Veronesi (FUV), via Solferino 19, 20121 Milano, Italy

## Abstract

Fragment-like molecules represent valuable starting points in anticancer drug discovery due to their structural simplicity and favorable physicochemical properties. In this context, the combination of phenotypic drug discovery (PDD) with fragment-based drug discovery (FBDD) offers an effective strategy for the rapid identification of bioactive scaffolds. Herein, a small in-house library of 6-chloropyridinyl benzoate fragments was designed to explore the antiproliferative potential of this underexplored scaffold. The compounds were first evaluated in silico, confirming favorable physicochemical and ADME profiles consistent with fragment-like chemical space, and were subsequently synthesized through simple one-pot procedures. Phenotypic screening against a panel of human cancer cell lines (A-375, HCT-116, and MCF-7) revealed a selective antiproliferative effect toward melanoma cells. Two derivatives, **7d** and **7k**, emerged as the most active compounds, displaying low micromolar activity against A-375 cells (IC_50_ = 1.59 ± 0.07 μM and 1.08 ± 0.03 μM, respectively) and high selectivity (SI > 60) compared to noncancerous 16-HBE cells. Mechanistic investigations demonstrated that these fragments induce apoptosis through an ROS-dependent mitochondrial pathway, associated with mitochondrial dysfunction and cell cycle arrest at the G1–S transition. Density functional theory (DFT) analysis further suggested that the enhanced electrophilic character and electron-accepting ability of the most active derivatives may contribute to their biological activity. Overall, this study identifies chloropyridinyl benzoate fragments as a previously unexplored scaffold with selective antimelanoma activity and highlights their potential as promising starting points for fragment-to-lead optimization.

## Graphical abstract

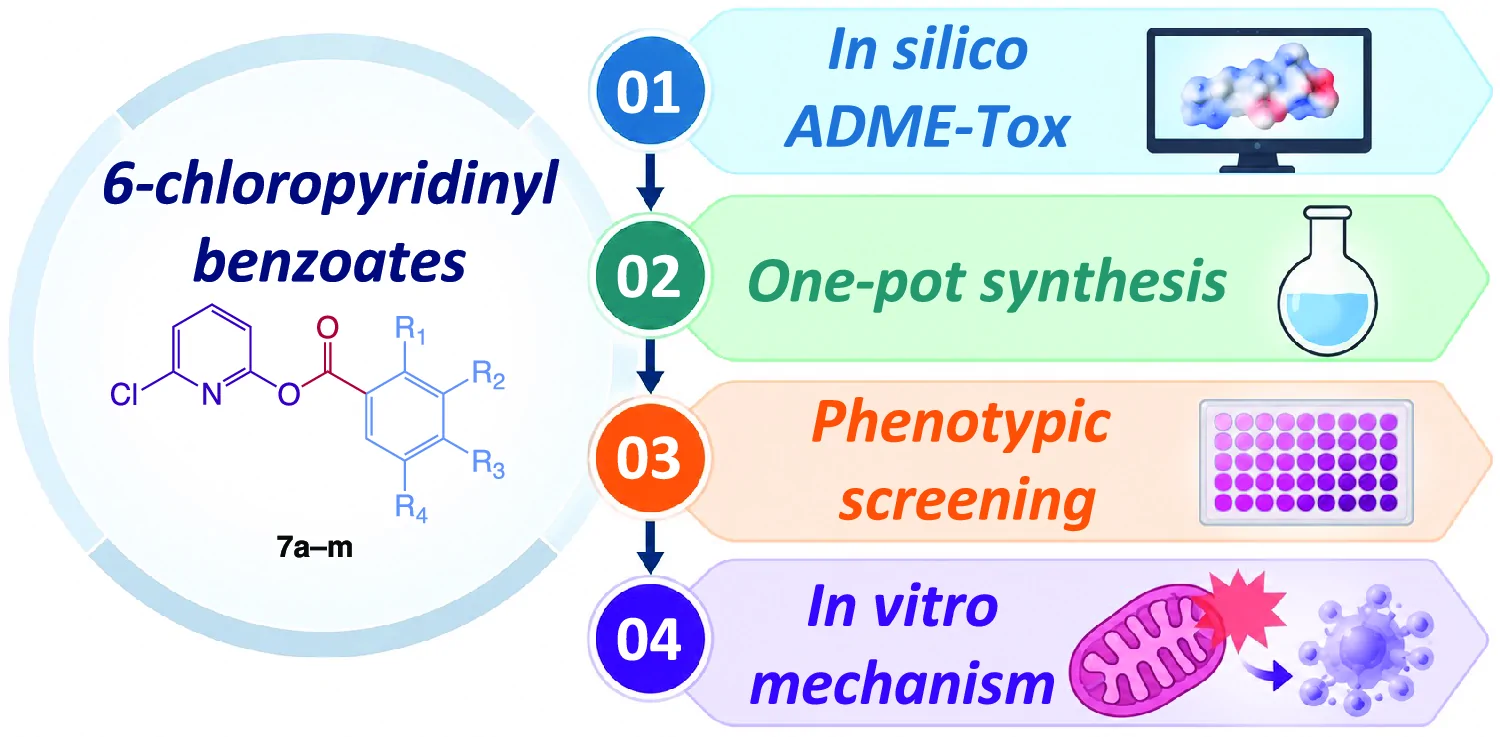

## Introduction

Phenotypic drug discovery (PDD) is based on the identification of bioactive compounds through the direct evaluation of their effects in cellular or organismal models, without prior knowledge of a specific molecular target. This approach enables the selection of compounds capable of eliciting a desired biological response within complex and physiologically relevant systems.^1^ Although PDD was partially overshadowed in the past by target-based strategies, it has recently regained interest due to its ability to capture the multifactorial nature of complex diseases such as cancer, whose underlying mechanisms are often not fully understood. In this context, phenotypic screening offers a significant advantage, as it allows the identification of active molecules in biologically relevant environments that better mimic disease complexity.^1^ An evolution of PDD approach is represented by its integration with fragment-based drug discovery (FBDD), a powerful complementary strategy that relies on the screening of libraries of small, low-molecular-weight compounds.^2–4^ Fragment-like molecules typically exhibit favorable physicochemical properties, including low molecular weight, moderate lipophilicity, reduced structural complexity, and high permeability. These features enable efficient exploration of chemical space, even with relatively small libraries, and facilitate the identification of novel scaffolds with modest activity (typically in the μM–mM range) that can serve as starting points for subsequent optimization into more potent bioactive lead compounds.^5–8^

Within this framework, pyridinyl aromatic esters have recently emerged as promising yet relatively underexplored fragment-like scaffolds. These compounds possess key characteristics of optimal fragments, including low molecular weight, appropriate reactivity, balanced lipophilicity, and favorable drug-like properties. The introduction of electron-withdrawing substituents, such as chlorine atoms on the pyridinyl ring, further enhances these properties, leading to chloropyridinyl derivatives with increased chemical versatility. Consistently, although still limited in number, several studies have highlighted the potential of this structural motif in different therapeutic contexts (Figure 1). In particular, fragment-like derivatives **1**–**3** have been identified as promising inhibitors of the main protease (Mpro) of SARS-CoV-2,^9–12^ supporting the suitability of pyridinyl ester scaffolds in fragment-based approaches. In addition, more complex molecules incorporating the same motif have been reported in anticancer research. Notably, compounds **4**–**6** represent kinase inhibitors active on lymphoma cells in which the pyridinyl ester moiety is embedded within more elaborated chemical architectures, further demonstrating its compatibility with structurally complex bioactive frameworks.^13–15^

**Figure 1:**
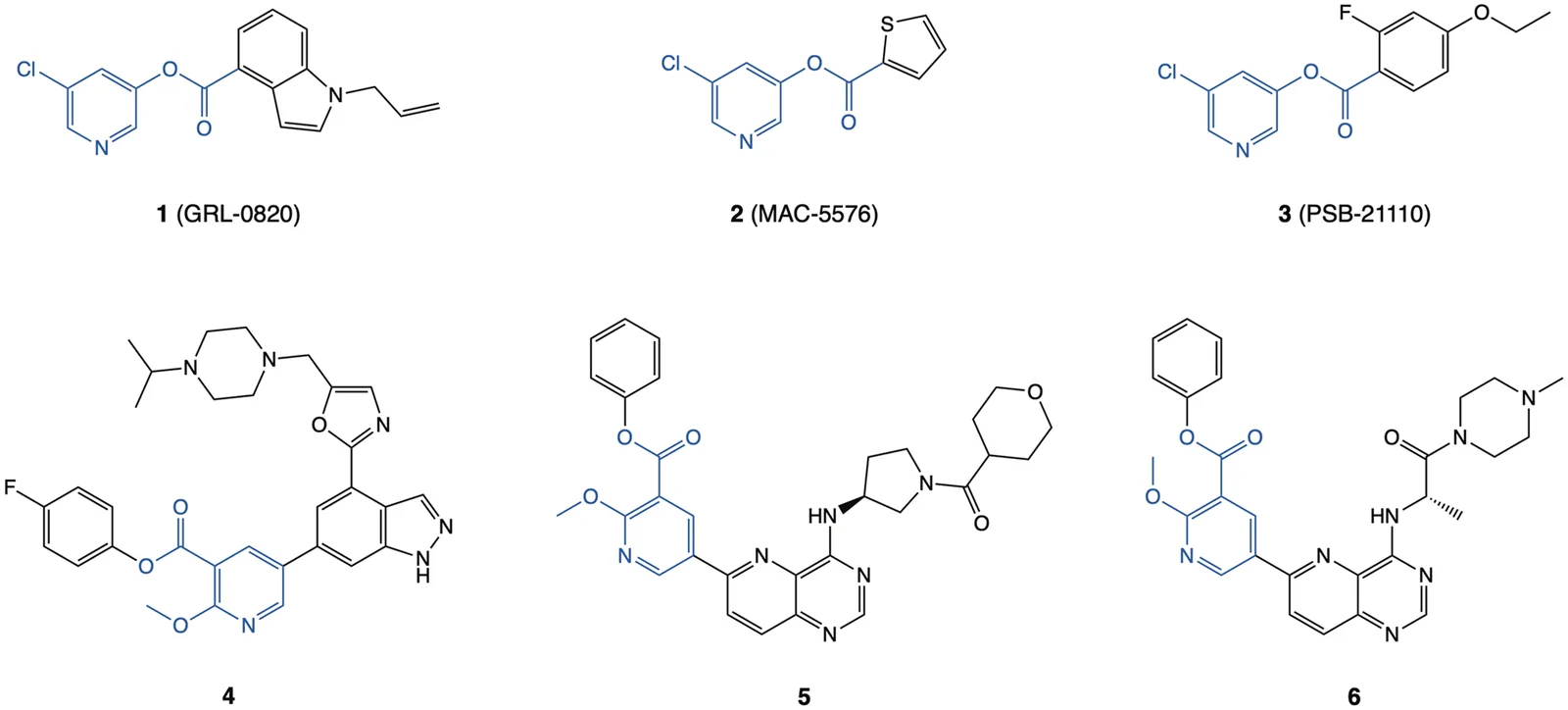
Representative examples of biologically active compounds (**1**–**6**) reported in the literature featuring the chloropyridinyl ester motif, ranging from fragment-like structures to more complex molecules, and associated with antiviral and anticancer activities.

Despite these considerations, to the best of our knowledge, a comprehensive phenotypic exploration of pyridinyl ester libraries in the context of anticancer activity has not yet been reported.

Based on these considerations, we designed and constructed a small in-house library of pyridinyl benzoate fragments bearing different substituents, in order to explore a broad chemical space. The fragment-like nature of the library was preliminarily assessed through in silico evaluation of both physicochemical and ADME properties. The synthesized compounds were then screened for their antiproliferative activity against a panel of human cancer cell lines to gain an overview of their biological profile. The most promising derivatives were further investigated to elucidate their mechanism of action, focusing on apoptosis induction, cell cycle modulation, and ROS production.

Finally, density functional theory (DFT) calculations were performed to provide insights into the electronic properties and reactivity of the most active compounds, supporting future fragment-to-lead optimization.

## Results and Discussion

### Physicochemical and ADME Analysis of the 6-Chloropyridinyl Benzoate In-House Fragment Library

The general chemical structure of the in-house 6-chloropyridin-2-yl benzoate fragment library and the substitution pattern defining compounds **7a**–**m** is reported in Figure 2. The small library herein presented was used to explore the structural diversity of this scaffold through systematic substitution on the benzoate ring. In total, 13 fragments were considered, bearing different substituents at positions R_1_–R_4_ of the phenyl ring.

**Figure 2:**
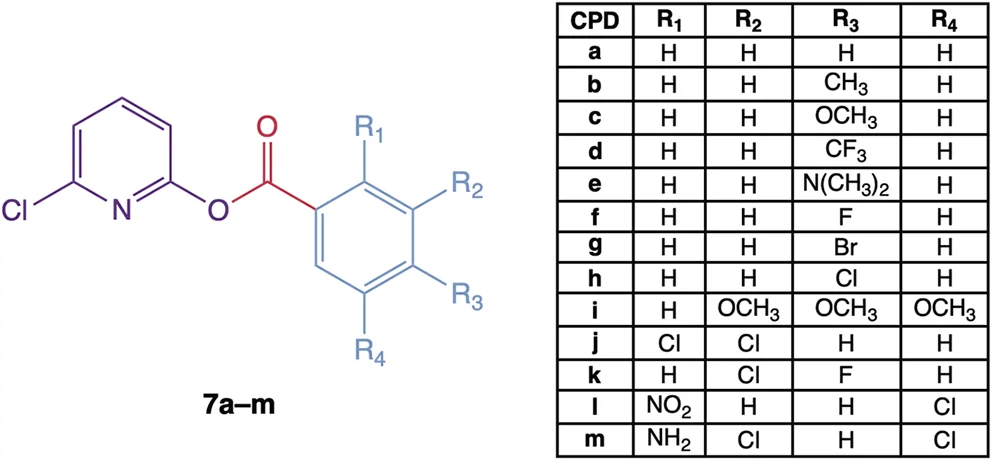
General structure of the in-house 6-chloropyridin-2-yl benzoate fragment library (**7a**–**m**). Substituents on the benzoate ring (R_1_–R_4_) include electron-donating and electron-withdrawing groups introduced to modulate the electronic properties of the scaffold.

Both electron-donating and electron-withdrawing groups were included to modulate the electronic properties of the aromatic system and to provide a chemically diverse fragment collection. Electron-donating groups (EDGs) included methyl, methoxy, dimethylamino and amino substituents, whereas fluoro, chloro, bromo, trifluoromethyl and nitro groups were selected as electron-withdrawing groups (EWGs). In addition, some derivatives bearing multiple substitutions on the phenyl ring were included to further broaden the structural variability within the series.

To obtain preliminary insights into the physicochemical profile and drug-likeness of the designed fragments, an in-silico analysis was performed using the SwissADME web server^16^ and QikProp^17^ calculations, enabling the estimation of key molecular descriptors and pharmacokinetic parameters related to ADME properties (see Experimental Section). The most relevant parameters are reported in Tables 1–3, while the complete set of calculated descriptors and plots is reported in the Supporting Information Tables S1 and S2 and Figure S1.

**Table 1:** Structural and Physicochemical Descriptors Predicted for the 6-Chloropyridin-2-yl Benzoate Derivatives **7a**–**m**

code	MW^a^	RB^a^	HBA^a^	HBD^a^	TPSA^a^	consensus LogP_o/w_ ^a^	ESOL LogS^a^
**7a**	233.65	3	3	0	39.19	2.87	–3.89
**7b**	247.68	3	3	0	39.19	3.29	–4.17
**7c**	263.68	4	4	0	48.42	2.94	–3.93
**7d**	301.65	4	6	0	39.19	4.00	–4.69
**7e**	276.72	4	3	0	42.43	2.96	–4.08
**7f**	251.64	3	4	0	39.19	3.26	–4.03
**7g**	312.55	3	3	0	39.19	3.49	–4.78
**7h**	268.10	3	3	0	39.19	3.49	–4.47
**7i**	323.73	6	6	0	66.88	2.93	–4.04
**7j**	302.54	3	3	0	39.19	3.87	–5.04
**7k**	286.09	3	4	0	39.19	3.79	–4.61
**7l**	313.09	4	5	0	85.01	2.88	–4.63
**7m**	317.56	3	3	1	65.21	3.60	–5.03

a MW: molecular weight; RB: rotatable bonds (optimal values in the range 0–15); HBA: hydrogen bond acceptor groups; HBD: hydrogen bond donor groups; TPSA: total polar surface area; LogP_o/w_: partition coefficient octanol/water in logarithmic scale; LogS: solubility in logarithmic scale.

The main structural and physicochemical properties of the designed compounds are summarized in Table 1. Overall, the analyzed parameters indicate that the library falls within the typical physicochemical space of bioactive fragments. In particular, the molecular weights (MW) range from 233.65 to 323.73 Da, while the number of rotatable bonds (RB) remains limited (3–6), indicating relatively compact and moderately flexible molecular structures. The hydrogen-bonding capacity is modest, with hydrogen bond acceptors ranging from 3 to 6 and hydrogen bond donors between 0 and 1, while the calculated TPSA values (39.19–85.01 Å2) indicate a polarity compatible with membrane permeability. Lipophilicity values are moderate across the series, with consensus LogP_(o/w)_ values between 2.87 and 4.00 (higher for halogen containing compounds) whereas the predicted aqueous solubility (ESOL LogS) ranges from −3.89 to −5.04.

These physicochemical descriptors indicate that the designed fragments occupy a chemical space compatible with fragment-like bioactive molecules. Based on these preliminary features, further in silico analyses were performed to evaluate their predicted ADME properties and overall drug-likeness.

In this context, the most relevant parameters related to the predicted pharmacokinetic profile of the investigated molecules are summarized in Table 2. Thanks to the optimal balance between lipophilicity and polarity, together with their relatively small molecular size, the compounds are predicted to exhibit very high Caco-2 permeability. This parameter is positively correlated with passive gastrointestinal (GI) absorption and is reflected in the predicted maximum percentage of human oral absorption (%HOA), which reaches 100% for almost all derivatives, with the only exception of compound **7l**, which contains a protonatable amine group. This favorable physicochemical balance is further confirmed by the predicted MDCK permeability values, which represent a widely used model for blood–brain barrier (BBB) permeation. Indeed, almost all compounds show high predicted MDCK permeability, suggesting a strong ability to cross even tight biological membranes and the BBB. Consistently, the SwissADME BOILED-Egg model^16,18^ (Figure 3) confirm graphically these results: most derivatives are located within the yolk region, indicating a high probability of both GI absorption and BBB permeation.

**Table 2:** Key In-Silico Predicted ADME Properties for Pyridinyl Benzoate Fragments **7a**–**m**

compound	QPPCaco^a^	%HOA	QPPMDCK	BBB permeant	P-gp substrate	QPlogKhsa	CYP2D6 inh.	CYP3A4 inh.
**7a**	2922.67	100	4008.37	yes	no	–0.196	no	no
**7b**	2920.66	100	4005.54	yes	no	–0.020	no	no
**7c**	2917.67	100	4001.21	yes	no	–0.220	no	no
**7d**	2916.43	100	10,000	yes	no	0.090	no	no
**7e**	2751.85	100	3755.77	yes	no	–0.054	no	no
**7f**	2922.65	100	7255.18	yes	no	–0.149	no	no
**7g**	2921.56	100	10000	yes	no	–0.042	no	no
**7h**	2922.33	100	9897.25	yes	no	–0.068	no	no
**7i**	2920.46	100	4004.94	yes	no	–0.252	no	no
**7j**	3096.05	100	10,000	Yes	no	–0.003	no	no
**7k**	2917.22	100	10,000	yes	no	–0.025	no	no
**7l**	620.45	92	1851.45	no	no	–0.223	no	no
**7m**	1309.23	100	9503.22	yes	no	0.170	no	no

a Abbreviations: HOA%, predicted Human Oral Absorption on 0 to 100% scale. BBB, blood-brain barrier. QPP-Caco2, predicted apparent Caco-2 cell permeability in nm/sec (<25 poor, >500 great). QPPMDCK, predicted apparent MDCK cell permeability in nm/sec (<25 poor, >500 great). QPlogKhsa, predicted binding to human serum albumin (optimal range −1.5 – 1.5). P-gp, glycoprotein P. CYP: cytochrome P450.

**Figure 3:**
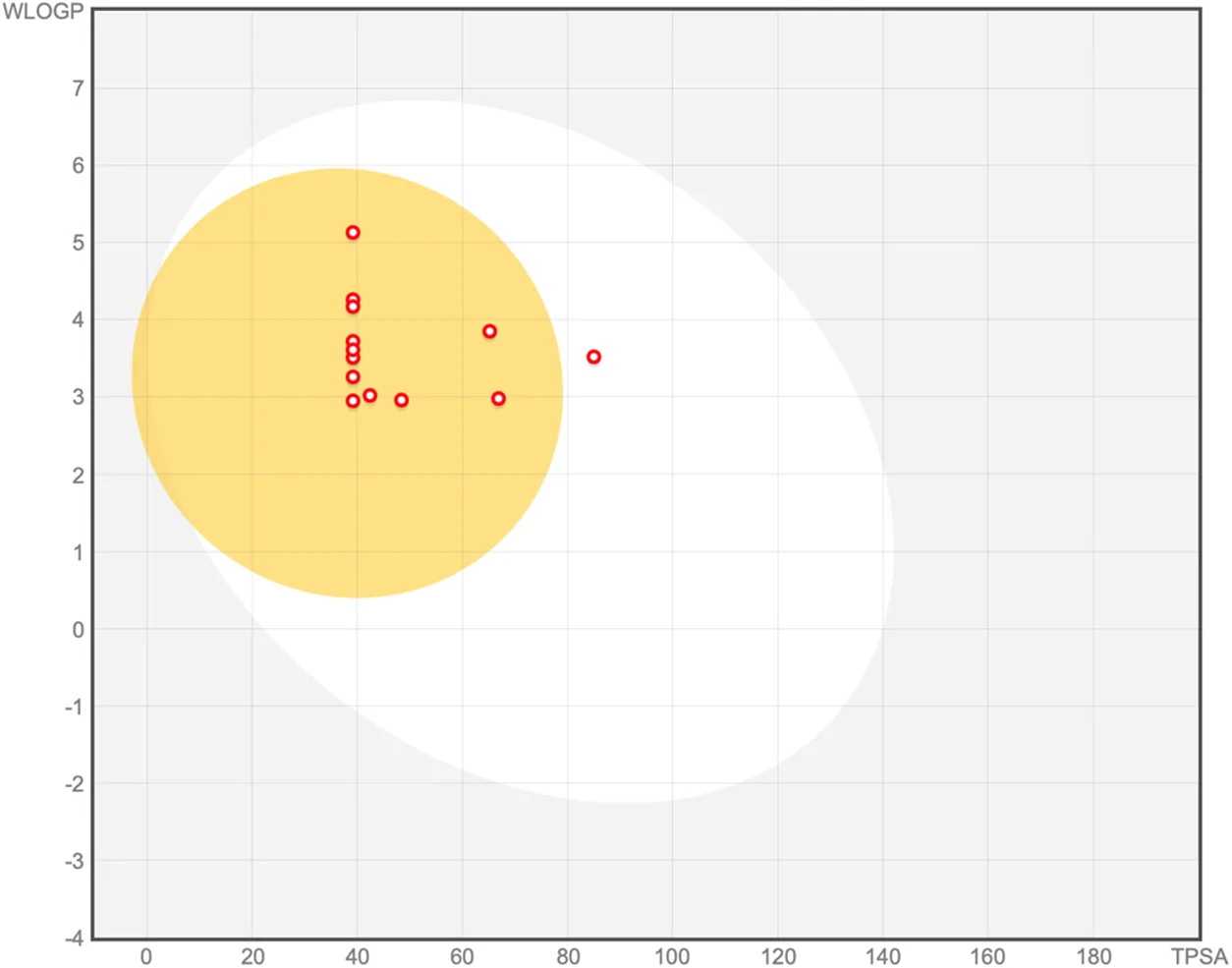
BOILED-Egg plot generated using the SwissADME tool for compounds **7a**–**m.** Molecules located in the white region are predicted to be passively absorbed in the gastrointestinal tract, whereas those falling within the yellow region (yolk) are predicted to penetrate the blood–brain barrier (BBB). Compounds positioned outside these regions are predicted to show low passive absorption and limited BBB permeability. Red dots indicate molecules that are not substrates of P-glycoprotein (Pgp).

With regard to distribution-related parameters, the investigated compounds were predicted to bind human serum albumin within an acceptable range of QPlogKhsa values, suggesting a balanced plasma protein binding profile that may support adequate systemic distribution.

Moreover, none of the compounds were predicted to behave as substrates of P-glycoprotein (P-gp), one of the best characterized efflux transporters that frequently contributes to reduced intracellular drug accumulation and drug resistance in cancer cells through active efflux mechanisms.

Regarding potential metabolic interactions, none of the investigated compounds were predicted to inhibit the major cytochrome P450 isoforms CYP2D6 and CYP3A4, which are responsible for the metabolism of a large fraction of xenobiotics.^19,20^ This result suggests a low likelihood of undesirable pharmacokinetic drug–drug interactions related to CYP inhibition.

Finally, the drug-likeness profile of the investigated compounds was further evaluated through the parameters summarized in Table 3. In particular, the QP stars descriptor, which indicates the number of calculated properties falling outside the range typically observed for known drugs, showed very low values across the series (0–2), suggesting that the investigated molecules largely fall within the chemical space associated with drug-like compounds. Moreover, none of the derivatives triggered alerts related to Lipinski’s rule of five (LRoF), as well as to other medicinal chemistry filters including Ghose (GV), Veber (VV), Egan (EV), and Muegge (MV) rules. In addition, no PAINS (pan-assay interference compounds) alerts were detected for any of the analyzed molecules.

**Table 3:** Drug-Likeness Parameters and Filters for Compounds **7a**–**m**

compound	QP stars^a^	LRoF	GV	VV	EV	MV	PAINS
**7a**	1	0	0	0	0	0	0
**7b**	0	0	0	0	0	0	0
**7c**	0	0	0	0	0	0	0
**7d**	2	0	0	0	0	0	0
**7e**	0	0	0	0	0	0	0
**7f**	1	0	0	0	0	0	0
**7g**	1	0	0	0	0	0	0
**7h**	1	0	0	0	0	0	0
**7i**	0	0	0	0	0	0	0
**7j**	2	0	0	0	0	0	0
**7k**	2	0	0	0	0	0	0
**7l**	0	0	0	0	0	0	0
**7m**	1	0	0	0	0	0	0

a Abbreviations: QP stars: number of property or descriptor values that fall outside the 95% range of similar values for known drugs (optimal values in the range 0–5). LRoF: Lipinski Rule of Five. GV: Ghose Violations. VV: Veber Violations. EV: Egan Violations. MV: Muegge Violations. PAINS: Pan-Assay Interference Compounds.

Overall, these results further support the favorable drug-like profile of all the investigated chloropyridinyl benzoate fragments and their suitability for subsequent synthesis and biological evaluation.

### Synthesis

The series of 6-chloropyridinyl ester fragments **7a**–**m** was prepared according to the two one-pot procedures outlined in Scheme 1. Following Route A, the target pyridinyl benzoates were obtained by reacting 2-chloro-6-hydroxypyridine (**8**) with commercially available benzoyl chlorides (**9a**–**k**) bearing different substituents. The high electrophilicity of the acyl chloride functionality enabled efficient ester formation with the relatively weakly nucleophilic phenolic group of **8**. The reactions were carried out in anhydrous DCM in the presence of pyridine as a base, affording the corresponding esters **7a**–**k** at room temperature within relatively short reaction times.

**Scheme 1:**
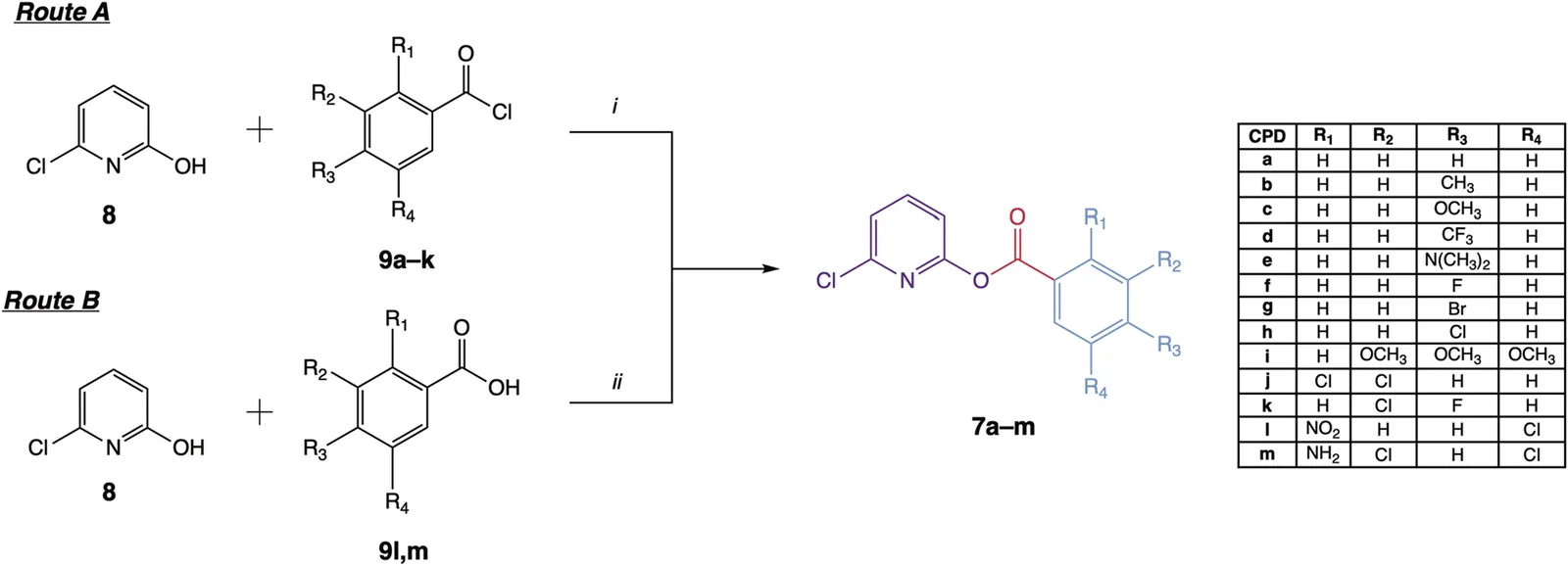
Synthesis of 6-Chloropyridinyl Ester Derivatives **7a**–**m^a^**

a Reagents and conditions: (i) 2-chloro-6-hydroxy-pyridine **8** (1 equiv), appropriate benzoyl chlorides **9a**–**k** (1.2 equiv), pyridine (1.2 equiv), anhydrous DCM, rt 12–24 h; (ii) 2-chloro-6-hydroxy-pyridine **8** (1 equiv), appropriate carboxylic acid **9l**,**m** (1 equiv), DCC (1.5 equiv), DMAP (0.5 equiv), N_2_, anhydrous DCM, rt 12–24 h.

Alternatively, Route B was employed for the preparation of compounds **7l**,**m** from the corresponding benzoic acids (**9l**,**m**). In this case, DCC was used as a carboxylic acid activating/dehydrating agent, while DMAP served as a basic acyl-transfer catalyst, enabling in situ activation of the carboxylic acids and subsequent esterification with 2-chloro-6-hydroxypyridine **8**. Both methods provided corresponding derivatives of type **7** in good to excellent yields (80–95%).

### Biology

#### Analysis of Cytotoxic Activity and Selectivity Index

To obtain preliminary insights into the antiproliferative potential of the synthesized fragment library, a PDD approach was performed. The complete set of drug-like 6-chloropyridinyl benzoate derivatives **7a**–**m** was tested at a single concentration (10 μM) for 24 h to evaluate the cytotoxicity against three human cancer cell lines representing different tumor types: A-375 (melanoma), HCT-116 (colorectal carcinoma), and MCF-7 (breast cancer), using the MTT assay. Table 4 presents selected growth percentages of treated cells, excluding values above 90%, which were not reported.

**Table 4:** One Dose (10 μM) Cytotoxic Activity of Compounds **7a**–**m** Against A-375 (Melanoma), HCT-116 (Colon), and MCF-7 (Breast) Cancer Cell Lines

	growth percent^a^		
compound	A-375	HCT-116	MCF-7
**7a**	67.3		
**7b**	89.1		
**7c**	56.2		88.8
**7d**	*20.1*		
**7e**	73.7		
**7f**	61.3		
**7g**	60.1	85.5	80.8
**7h**	77.3	80.1	83.9
**7i**	52.5	87.2	89.7
**7j**	83.2	79.8	86.2
**7k**	*10.8*	87.2	
**7l**	79.9		
**7m**	58.3	79.9	85.3

a Cells were treated with the compounds for 24 h, and cell viability was assessed using the MTT assay, with vehicle-treated cells serving as the control, as described in Section 5.2. Data are expressed as the mean ± SD from three independent experiments performed in triplicate. Italicized data indicate cell growth values below the 50% viability threshold.

Analysis of the single-dose screening results revealed that the tested compounds did not exhibit significant antiproliferative activity against the HCT-116 and MCF-7 cell lines. Only a few derivatives showed growth percentages (G%) below 90%, and none reduced cell viability below 80%, indicating a generally low sensitivity of these two cancer models to the investigated scaffold.

Conversely, more promising results were obtained for the melanoma cell line A-375, suggesting a certain degree of selectivity of this chemical series toward melanoma cells. In this context, a preliminary structure–activity relationship (SAR) analysis could be inferred based on the substitution pattern of the benzoyl moiety.

In particular, compounds **7d** and **7k**, bearing a para-CF_3_ and a 3-Cl-4-F substituent, respectively, emerged as the most active derivatives. Both molecules are characterized by multiple halogen substituents (especially fluorine) and were able to markedly inhibit A-375 cell growth, producing G% values of 20.1 and 10.8, corresponding to approximately 80 and 90% growth inhibition, respectively. A second group of compounds, including **7a** (unsubstituted), **7c** (p-OCH_3_), **7f** (p-F), **7g** (p-Br), **7i** (3,4,5-OCH_3_) and **7m** (2-NH_2_-3,5-Cl), displayed moderate activity, with G% values ranging between 50 and 70%. On the other hand, derivatives **7b** (p-CH_3_), **7e** (p-N­(CH_3_)_2_), **7h** (p-Cl), **7j** (2,3-Cl) and **7l** (2-NO_2_-5-Cl) showed weak antiproliferative effects, with growth percentages between 70 and 90%.

Overall, these results suggest that the presence of halogen substituents (especially fluorinated) with moderate electron-withdrawing character, particularly when combined in multihalogenated patterns, may contribute to improved antiproliferative activity within this series. To the best of our knowledge, small chloropyridinyl benzoate fragments have not previously been explored in the context of melanoma-targeted antiproliferative agents, and the present results suggest that this scaffold may warrant further investigation.

Thus, using a threshold criterion of 50% maximum cell viability, compounds **7d** and **7k** were selected as the most active in inhibiting cell growth for further analysis. Their cytotoxic activity was further investigated in A-375 cells, which were the most sensitive to the tested compounds, in order to determine the IC_50_ values. When tested over a range of 0.1–10 μM for 24 h, both compounds inhibited A-375 cell growth in a concentration-dependent manner (Figure 4), with IC_50_ values of 1.59 ± 0.07 μM and 1.08 ± 0.03 μM for compounds **7d** and **7k**, respectively.

**Figure 4:**
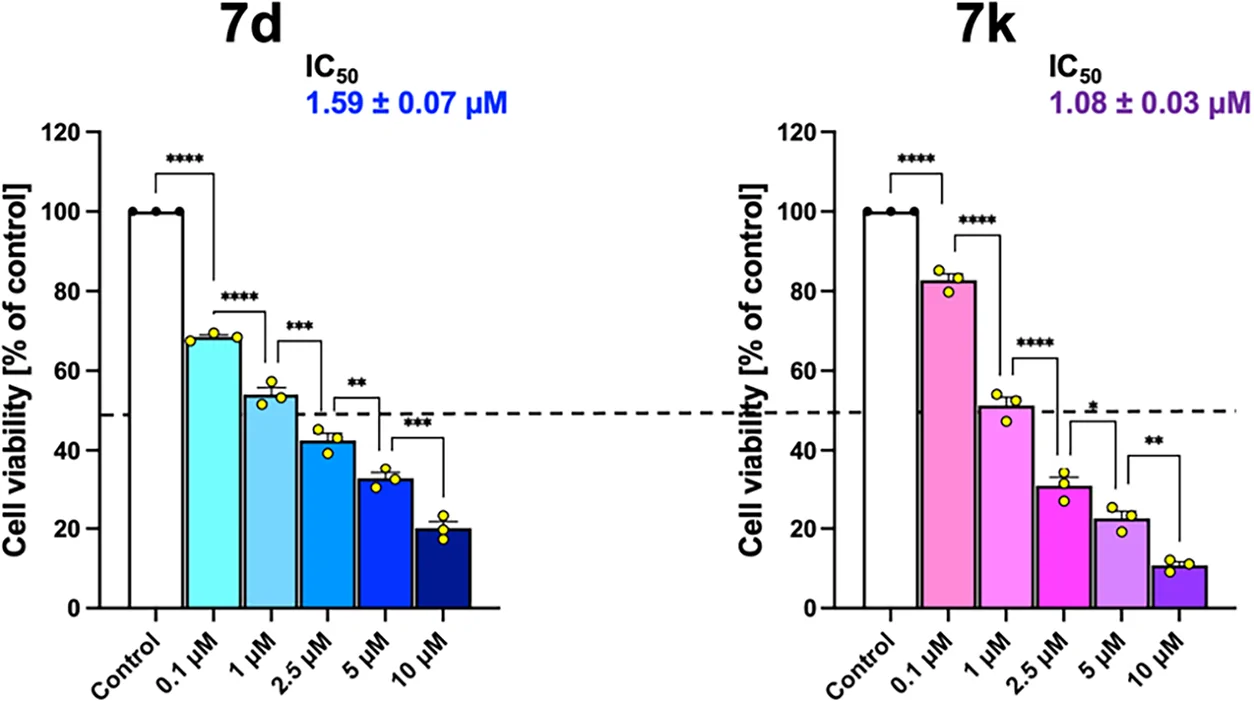
Antiproliferative activity of pyridinyl fragments **7d** and **7k** in A-375 cells. Cells were treated with increasing concentrations (0.1, 1, 2.5, 5, and 10 μM) for 24 h, and cell viability was determined using the MTT assay. The dashed line indicates 50% cell viability (IC_50_ threshold). *p < 0.05, **p < 0.01, ***p < 0.005, ****p < 0.001 (ANOVA associated with Tukey’s test). Values are the mean ± SD of four separate experiments conducted in triplicates.

Moreover, the inhibitory effect of the selected compounds on cell growth was assessed in noncancerous 16-HBE bronchial epithelial cells to determine the selectivity index (SI) relative to cancer cells.^21^ Cells were treated with both compounds over a concentration range of 0.1–100 μM for 24 h. Notably, 16-HBE cell viability was only moderately affected after 24 h of exposure to compounds **7d** and **7k**. At the highest tested concentration (100 μM), compounds **7d** and **7k** reduced cell viability by approximately 38 and 33%, respectively (Figure 5). Although exact IC_50_ values could not be determined for the normal cells, it can be confidently estimated that the SI value for both compounds exceeds 62. This estimation is based on the conservative assumption that the IC_50_ in 16-HBE cells equals the highest concentration tested (100 μM); under this scenario, the selectivity index for the less potent compound (compound **7d**, with an IC_50_ of 1.59 μM in A-375 cells) would still be 62.8.

**Figure 5:**
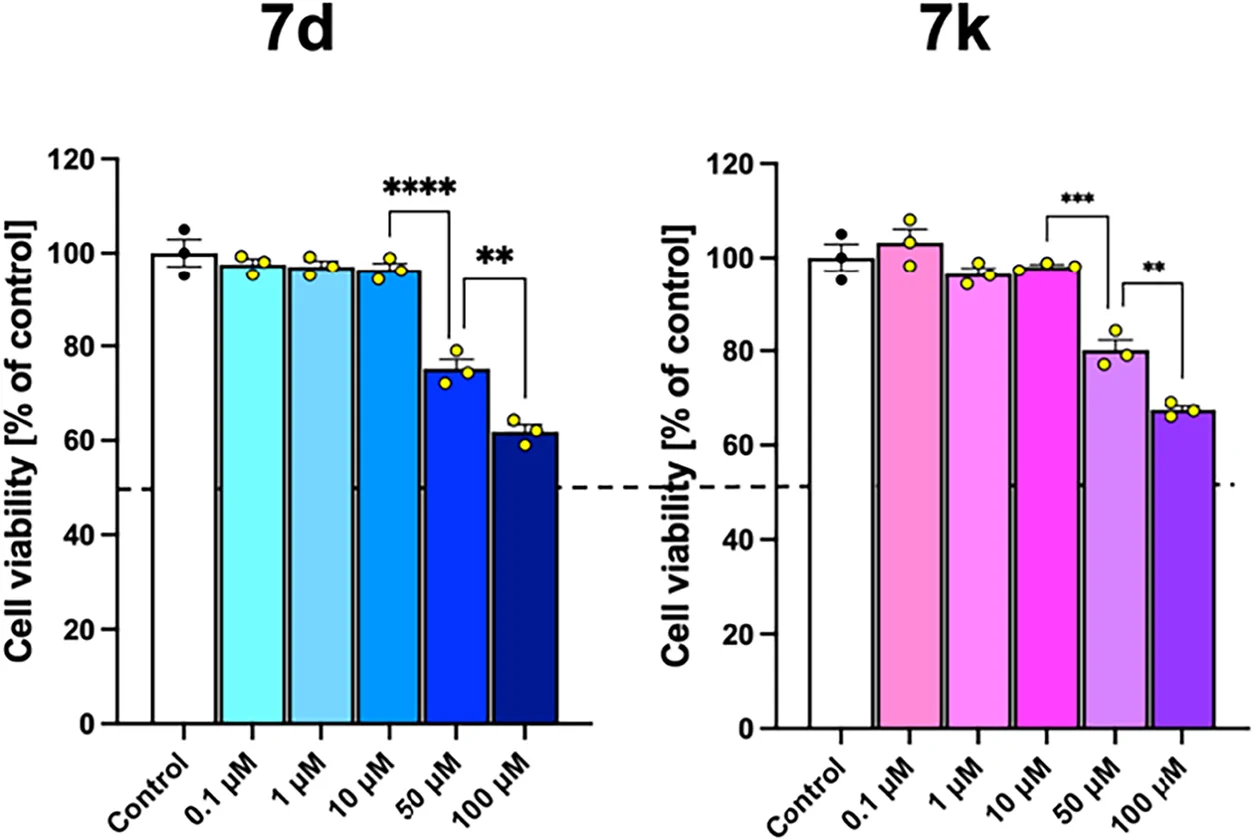
Antiproliferative activity of pyridinyl fragments **7d** and **7k** in 16-HBE cells. Cell viability was assessed after 24 of treatment by MTT assay. * **p < 0.01, ***p < 0.005, ****p < 0.001 (ANOVA associated with Tukey’s test). Values are the mean ± SD of four separate experiments conducted in triplicates.

An SI above 10 is generally taken as evidence of meaningful selectivity in vitro.^22^ Both **7d** and **7k** clearly exceed this value. They were strongly cytotoxic to A-375 cells and had only a limited effect on 16-HBE cells. Current melanoma therapies often carry off-target toxicity.^23,24^ A scaffold that spares nontransformed cells is therefore of interest, because the therapeutic window of an anticancer agent depends largely on this property.^25^ Given the potency and selectivity observed at 24 h, with **7k** slightly more active than **7d**, we used A-375 cells to study the mechanism behind the antiproliferative effect of both fragments.

#### Pyridinyl Fragments **7d** and **7k** Induce Apoptosis Through a ROS-Dependent Mitochondrial Intrinsic Pathway in A-375 Cells

Annexin V-FITC/PI double staining was used to distinguish apoptotic from necrotic death in A-375 cells. Annexin V binds phosphatidylserine exposed on the outer membrane leaflet during apoptosis. PI enters only cells that have lost membrane integrity. Together, the two probes separate early apoptotic, late apoptotic and necrotic populations.^26^ In this regard, A-375 cells were treated for 24 h with the two effective derivatives, assayed at concentrations corresponding to half of the IC_50_ (IC_25_) and the IC_50_. Specifically, IC_50_ concentrations of 1.60 μM and IC_25_ of 0.8 μM were tested for compound **7d**, and 1.08 μM and 0.55 μM for compound **7k**. The data, shown in Figure 6A, indicated that none of the assayed fragments induced significant necrosis in A-375 cells, as evidenced by the low number of necrotic cells, which were 1.43 and 1.07% for **7d**, and 1.35 and 1.06% for **7k**, compared to 0.74% in control cells. Conversely, a concentration-dependent alteration in the cellular state was observed, accompanied by a progressive and significant increase in early apoptotic events (Figure 6A). The percentage of annexin V-FITC-positive cells increased in function of the concentration of both compounds, reaching 15.84 ± 1% and 19.0 ± 2.2% for **7d**, and 17.11 ± 1.14% and 23.52 ± 2.12% for **7k** at IC_25_ and IC_50_, respectively, compared to 5.95 ± 0.65% in control cells (*p* < 0.001, Figure 6B). Both **7d** and **7k** therefore killed A-375 cells mainly by apoptosis, and the apoptotic fraction grew with concentration. Necrotic cells remained close to control levels. This profile is desirable for an anticancer lead. Apoptotic cells are dismantled in an orderly way and cleared without releasing their content. Necrosis instead ruptures the plasma membrane and spills intracellular material, which can drive local inflammation and damage the surrounding tissue.^27^

**Figure 6:**
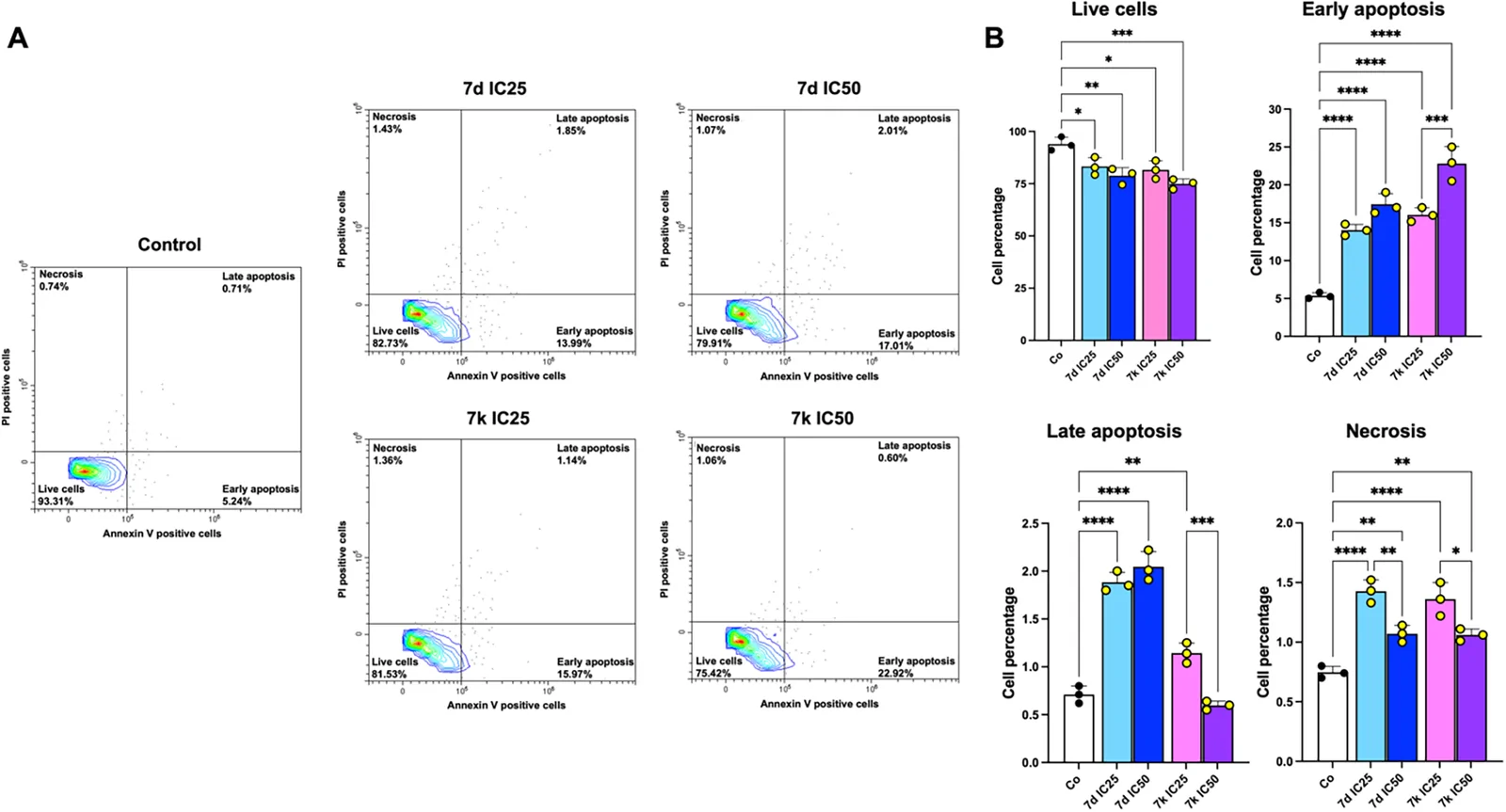
Apoptotic effect of **7d** and **7k** in A-375 cells, quantified by phosphatidylserine externalization. Cells were treated for 24 h with IC_25_ and IC_50_ of compounds and percentage of AnnexinV-FITC/PI double stained A-375 cells were determined by flow cytometer and compared to untreated cells (control). (A) Representative flow cytometry plots showing: viable cells (Annexin V^–^/PI^–^), early apoptotic cells (Annexin V^+^/PI^–^), late apoptotic cells (Annexin V^+^/PI^+^), and necrotic cells (Annexin V^–^/PI^+^). (B) Quantitative data are expressed as mean values ± SD of three separate experiments in triplicate. *p < 0.05, **p < 0.01, ***p < 0.005, ****p < 0.001 (one-way Anova associated with Tukey’s post hoc test).

Loss of ΔΨm is an early marker of mitochondrial damage.^28^ Depolarization of the inner membrane accompanies the release of cytochrome c and other pro-apoptotic factors, which commits the cell to the intrinsic pathway.^29^ We measured ΔΨm by flow cytometry with DiOC6, a cationic dye that accumulates in mitochondria in proportion to their membrane potential.^30^ The results showed that both compounds induced a significant, concentration-dependent decrease in DiOC6 uptake in A-375 cells after 24 h of treatment, indicating mitochondrial permeability dysfunction. Specifically, a 4.3 and 6.2-fold increase in mitochondrial depolarization was measured for compound **7d** and 5.2- and 7.8-fold for **7k**, at the corresponding IC_25_ and IC_50_ concentrations, respectively (Figure 7A), compared to untreated control cells (*p* < 0.001, Figure 7B). These findings suggest that both derivatives induced significant mitochondrial dysfunction, triggering the intrinsic mitochondrial pathway and contributing to the apoptotic effects observed in A-375 cells.

**Figure 7:**
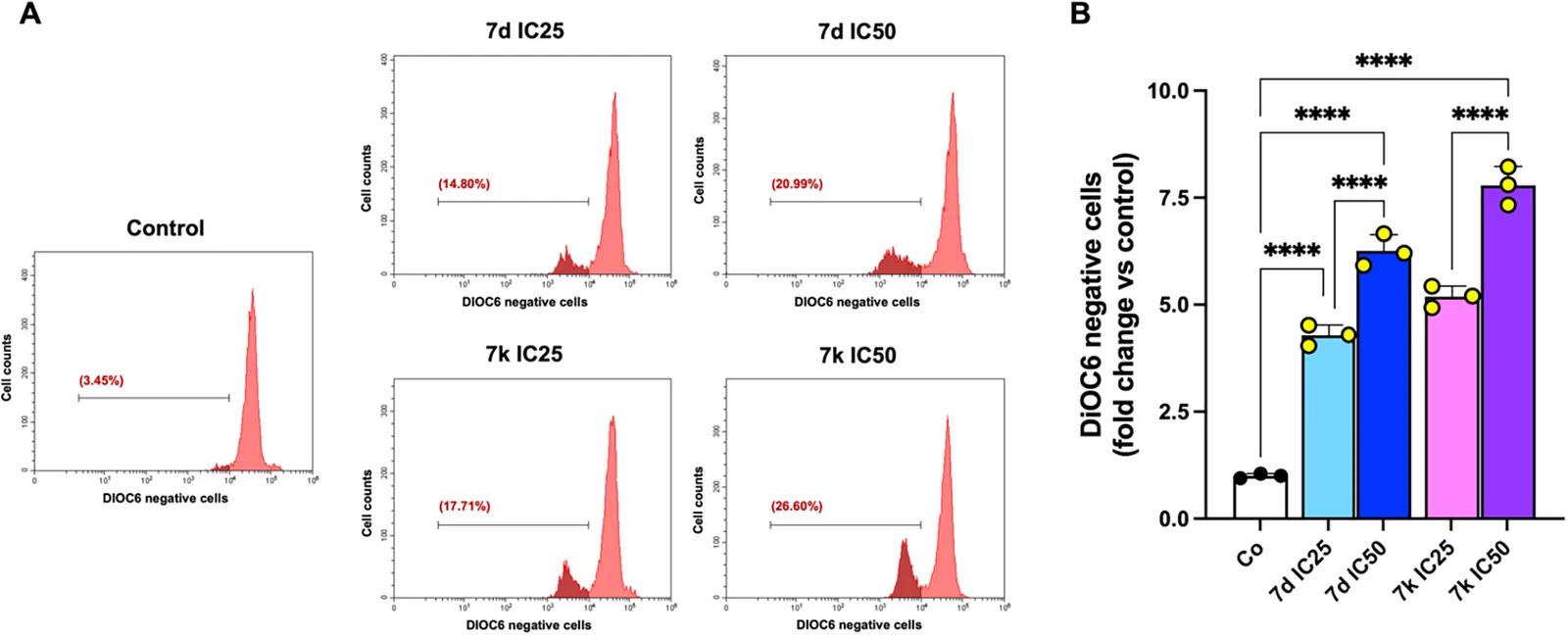
Loss of mitochondrial membrane potential induced by ester derivatives **7d** and **7k** in A-375 cells. Cells were treated for 24 h with IC_25_ and IC_50_ of compounds. Then the percentages of DiOC6 negative-A375 cells were determined by a flow cytometer and compared to untreated cells (control). (A) Representative flow cytometric images. (B) Mean values ± SD of three separate experiments in triplicate. ****p < 0.001 (one-way Anova associated with Tukey’s post hoc test).

Mitochondrial damage and redox imbalance reinforce each other, and both can push cells toward apoptosis.^31,32^ We therefore asked whether **7d** and **7k** raised intracellular ROS levels. Flow cytometric analysis using the fluorescent probe DCFDA revealed a significant, concentration-dependent increase in ROS levels in A-375 cells after 24 h of treatment, as shown in Figure 8A. In particular, ROS production increased by 2 and 3.8-fold following exposure to compound **7d**, and by 2.8 and 4.4-fold for **7k**, at concentrations equivalent to IC_25_ and IC_50_, respectively, compared to untreated control cells (*p* < 0.001 Figure 8B). Depolarized mitochondria leak electrons from the respiratory chain and become a major source of ROS. Sustained ROS accumulation damages lipids, proteins and DNA and activates apoptotic signaling.^33–35^ The parallel rise in ROS and loss of ΔΨm therefore points to oxidative stress as a central component of the cytotoxic mechanism of **7d** and **7k**.

**Figure 8:**
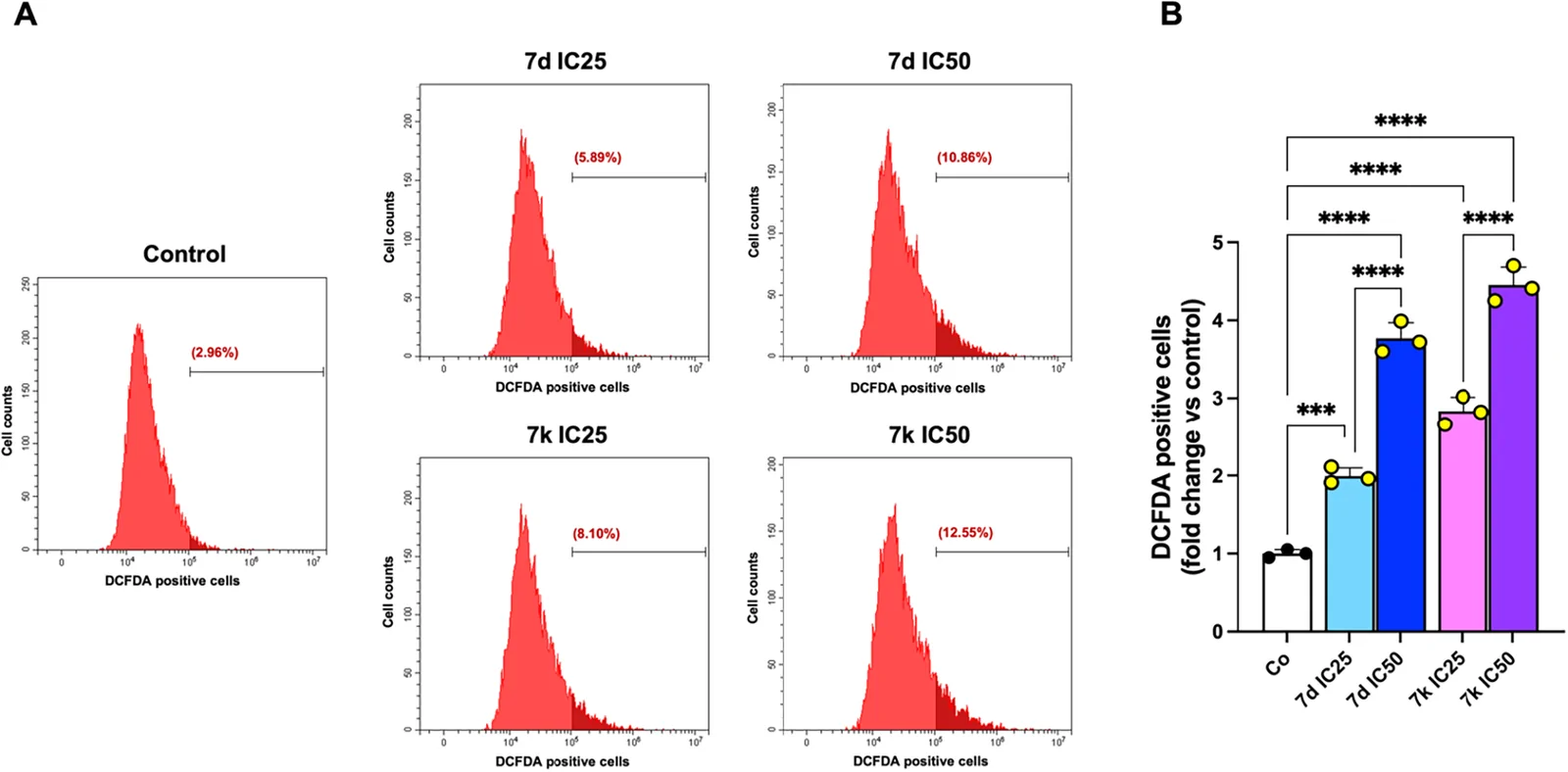
Effect of ester derivatives **7d** and **7k** on intracellular ROS production in A-375 cells. Cells were treated for 24 h with IC_25_ and IC_50_ of compounds and ROS levels were evaluated using flow cytometry after 24 h of treatment by staining of the cells with DCFDA as described in Section 5.2. Data are compared to untreated cells (control). (A) Representative flow cytometric images. (B) Mean values ± SD of three separate experiments in triplicate. ****p < 0.001 (one-way Anova associated with Tukey’s post hoc test).

#### Compounds **7d** and **7k** Induce Cell Cycle Arrest at the G1-S Phase Transition in A-375 Cells

Cancer cells escape the checkpoints that normally restrain division. Compounds that restore a block at a defined phase are therefore of interest as antitumor agents. We analyzed DNA content by PI staining and flow cytometry in A-375 cells treated with **7d** and **7k** for 24 h. Treatment with these compounds led to a significant accumulation of A-375 cells at the G1-S transition, accompanied by a decrease in the cellular population in the G2-M phase. Notably, exposure to the pyridinyl derivatives at IC_25_ and IC_50_ concentrations led to a moderate but statistically significant increase in the G0/G1 phase population: 60.9 ± 2.6% and 64.8 ± 2.2% for compound **7d**, and 61.1 ± 2.9% and 65.3 ± 4.4% for compound **7k**, compared to 56.9 ± 2.6% in control cells (*p* < 0.05 for both compounds at IC_50_, Figure 9). Simultaneously, treatment with the same compounds induced an accumulation of cells in the S phase, reaching 15.6 ± 0.8% and 20.4 ± 1.1% for compound **7d**, and 15.8 ± 0.9% and 20.7 ± 1.5% for compound **7k** at IC_25_ and IC_50_ concentrations, respectively, compared to 13.3 ± 0.5% in control cells (*p* < 0.001 for both compounds at IC_50_, Figure 9). Furthermore, a dose-dependent decrease in the G2/M phase population was observed following treatment: 24.3 ± 0.7% and 16.1 ± 0.6% for compound **7d**, and 23.4 ± 1.6% and 14.3 ± 0.7% for compound **7k** at IC_25_ and IC_50_ concentrations respectively, compared to 30.1 ± 0.9% in control cells (*p* < 0.005 for compound **7d** at IC_25_, and *p* < 0.001 for compound **7d** at IC_50_ and compound **7k** at both IC_25_ and IC_50_, Figure 9). This alteration of cell cycle progression, associated with the arrest of cells in the G1-S transition, significantly contributed to the overall antiproliferative activity of the investigated fragments. Furthermore, the imbalance of cellular redox homeostasis induced by the treatment may have triggered apoptosis through the activation of a ROS-dependent signaling pathway, resulting in cell cycle block. The accumulation of cells in S-phase suggests DNA replication stress, potentially triggered by cellular damage induced by our compounds or impaired DNA synthesis. This condition may activate intra-S-phase checkpoints, leading to cell cycle arrest and, if the damage remains unresolved, apoptotic signaling. Moreover, the slight increase in the cell population in the G0-G1 phase, observed following treatment with ester derivatives, suggests that this effect may influence the early stages of the cell cycle, likely through the activation of regulatory mechanisms in response to cellular stress, aimed at preventing cell cycle progression under unfavorable conditions.

**Figure 9:**
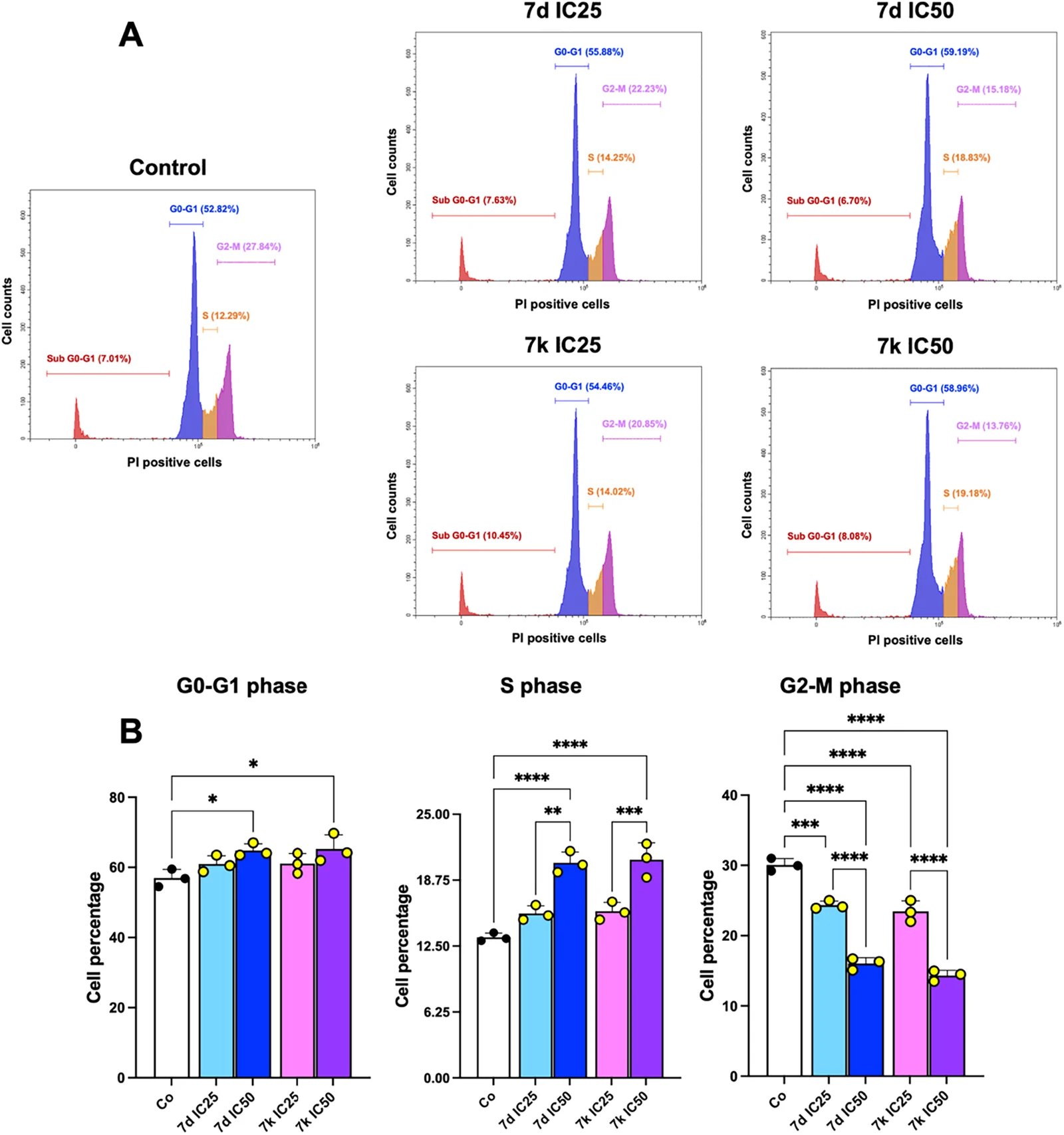
Effect of pyridinyl fragments **7d** and **7k** on the distribution of cell cycle phases in A-375 cells. Cells were treated for 24 h with IC_25_ and IC_50_ of compounds. Then samples were submitted to flow cytometry analysis after PI staining and cell distribution compared to untreated cells (control). (A) Representative flow cytometric images. (B) Each bar shows the percentage of viable cells in the different phases. *p < 0.05, **p < 0.01, ***p < 0.005, ****p < 0.001 (one-way Anova associated with Tukey’s post hoc test).

### DFT-Based Electronic Structure Analysis

To gain insight into whether the different substituents could influence the electronic properties of the molecules, and consequently their reactivity and biological activity, density functional theory (DFT) calculations were performed on representative compounds of the investigated series. To this end, in addition to the two most active derivatives, **7d** and **7k** (bearing p-CF_3_ and 3-Cl-4-F substituents, respectively), two further compounds were selected as representative examples of fragments displaying moderate and negligible activity. In particular, compound **7f** (p-F) was chosen as a representative of moderately active derivatives (50 < G% < 70), while compound **7b** (p-CH_3_) was selected as an example of inactive compounds (G% > 70).

Geometry optimizations were carried out at the B3LYP/6–31G** level of theory,^36,37^ and the energies of the frontier molecular orbitals were used to derive a set of global reactivity descriptors according to Koopmans’ approximation.

The calculated values of the HOMO and LUMO energies, together with the derived global descriptors including ionization potential (IP), electron affinity (EA), chemical potential (μ), electronegativity (χ), chemical hardness (η), global softness (S), and electrophilicity index (ω), are summarized in Table 5. All orbital energies are reported in Hartree units.

**Table 5:** Calculated Global Reactivity Descriptors for Representative Compounds **7b**, **7f**, **7d**, and **7k**, Including HOMO, LUMO, Energy Gap, Ionization Potential (IP), Electron Affinity (EA), Chemical Potential (μ), Electronegativity (χ), Chemical Hardness (η), Global Softness (S), and Electrophilicity Index (ω), Derived from HOMO and LUMO Energies According to Koopmans’ Approximation^a^

CPD	E_HOMO_	E_LUMO_	gap	IP	EA	μ	χ	η	S	ω
**7b**	–0.256125	–0.060267	0.1959	0.2561	0.0603	–0.1582	0.1582	0.0979	5.10	0.1278
**7d**	–0.267457	–0.075367	0.1921	0.2675	0.0754	–0.1714	0.1714	0.0961	5.21	0.1529
**7f**	–0.261953	–0.061940	0.2000	0.2620	0.0619	–0.1620	0.1620	0.1000	5.00	0.1311
**7k**	–0.263986	–0.068433	0.1956	0.2640	0.0684	–0.1662	0.1662	0.0978	5.11	0.1412

a Energies are expressed in Hartree.

Inspection of the calculated frontier orbital energies reveals that the most active derivatives (**7d** and **7k**) exhibit slightly lower LUMO energies compared with the less active analogues (**7b** and **7f**). Since the LUMO energy reflects the ability of a molecule to accept electrons, this trend suggests an increased electrophilic character for the most active compounds. Consistently, the calculated electrophilicity index (ω) shows a modest increase for compounds **7d** and **7k**, indicating a greater propensity to interact with electron-rich environments.

The HOMO–LUMO energy gaps remain relatively similar across the investigated series, indicating comparable overall electronic stability among the derivatives. However, the slightly smaller gap observed for compound **7d** and **7k** suggests a marginally higher chemical reactivity, which may contribute to enhanced antiproliferative activity.

The spatial distribution of the frontier molecular orbitals further supports this interpretation. As shown in Figure 10, the LUMO orbitals of the most active compounds (**7d** and **7k**) are mainly localized in the region of the ester carbonyl and the substituted benzoyl moiety, highlighting an electron-deficient region within the molecular scaffold. The enhanced electrophilic character and lower LUMO energies of compounds **7d** and **7k** may facilitate electron-transfer processes within the cellular environment, potentially contributing to the ROS overproduction and oxidative stress observed in the biological experiments. This electronic feature may favor interactions with nucleophilic sites present in biological macromolecules and promote oxidative stress within the cellular environment. In addition, the increased electrophilic character of the most active derivatives might also enable transient interactions with nucleophilic residues of biomolecular targets, which could contribute to the enhanced biological activity observed for these compounds.

**Figure 10:**
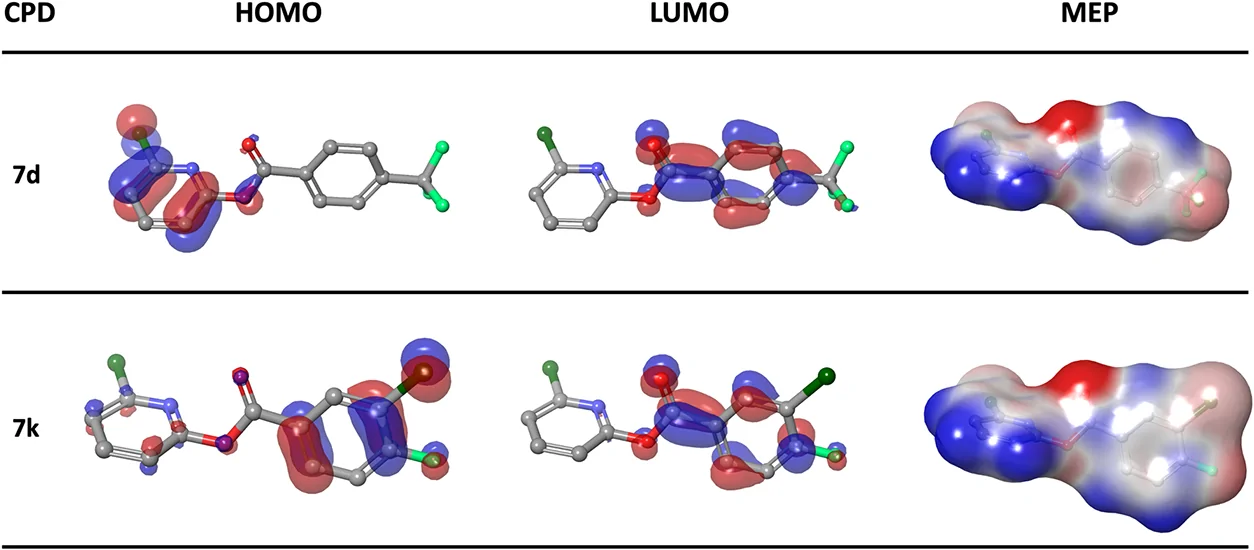
Frontier molecular orbitals (HOMO and LUMO) and molecular electrostatic potential (MEP) maps of the most active compounds **7d** and **7k** obtained from DFT calculations. HOMO and LUMO orbitals are represented as colored isosurfaces, while the MEP is mapped onto the electron density surface. In the MEP representation, red regions indicate areas of negative electrostatic potential (electron-rich), whereas blue regions correspond to positive electrostatic potential (electron-deficient).

Overall, the DFT analysis suggests that the electronic properties of the chloropyridinyl benzoate scaffold, particularly the electrophilic character associated with the ester functionality, may play a role in modulating the antiproliferative activity of this fragment series.

## Conclusions

The combination of phenotypic drug discovery (PDD) with fragment-based drug discovery (FBDD) represents an effective strategy for the rapid identification of small molecules with favorable physicochemical properties and potential biological activity.^1,4–6^ Within this framework, pyridinyl ester derivatives are emerging as promising and still underexplored fragment-like scaffolds, showing increasing relevance in different therapeutic areas.

In this study, we investigated the antiproliferative potential of a small in-house library of 13 chloropyridinyl benzoate fragments bearing diverse substituents on the aromatic ring, designed to explore a broad chemical space. In silico evaluation confirmed their favorable physicochemical and ADME properties, supporting their suitability as fragment-like molecules. The compounds were synthesized through two simple one-pot procedures, enabling the rapid generation of the derivative set, a key advantage in fragment-based drug discovery approaches.

Phenotypic antiproliferative screening identified two fragments, **7d** and **7k**, as the most active compounds, displaying low micromolar activity against human melanoma A-375 cells. Notably, both compounds showed high selectivity compared to other cancer cell lines and noncancerous cells, indicating a preferential cytotoxic effect toward melanoma cells. To the best of our knowledge, this represents the first report of chloropyridinyl benzoate fragments exhibiting selective antiproliferative activity against melanoma cells.

A comprehensive biological evaluation was performed to further characterize their activity, including apoptosis analysis, cell cycle distribution, and mitochondrial function assessment. These studies demonstrated that the most active fragments induce apoptosis through a ROS-dependent mitochondrial pathway, accompanied by measurable alterations in cell cycle progression.

In addition, density functional theory (DFT) calculations provided further insights into the electronic properties of the most active derivatives, highlighting features associated with increased electrophilicity and electron-accepting ability, which may contribute to their biological activity. These findings highlight, for the first time, the potential of chloropyridinyl benzoate fragments as antiproliferative agents and support their further development as promising scaffolds for anticancer drug discovery.

In conclusion, the application of a phenotypic drug discovery (PDD) strategy combined with fragment-based drug discovery (FBDD) to a small library of chloropyridinyl benzoate fragments enabled the identification of derivatives with significant and selective antiproliferative activity against A-375 melanoma cells, well-known for their aggressiveness and invasiveness. The obtained results suggest that the electronic features of this scaffold, including its potential electrophilic character, may contribute to its biological activity, supporting further investigation of a possible electrophilic reactivity profile.

Overall, chloropyridinyl benzoate fragments represent valuable starting scaffolds for fragment-to-lead optimization (e.g., growing and merging strategies) toward more potent and selective antimelanoma agents, warranting further mechanistic and in vivo studies.

## Experimental Section

### Chemistry

#### General Experimental Procedures

Unless otherwise specified, commercially available reagents and solvents were used as received. Melting points were measured in open capillary tubes using a Büchi Tottoli apparatus and are reported without correction. IR spectra were recorded using a Jasco FT/IR-5300 spectrophotometer. ^1^H and ^13^C NMR spectra were acquired at 400 and 100 MHz, respectively, on a Bruker AC-E spectrometer using CDCl_3_ or DMSO-d_6_ as solvents. Chemical shifts are reported in ppm relative to tetramethylsilane (TMS). Signal multiplicities are abbreviated as follows: br s, s, d, t, q, and m for broad singlet, singlet, doublet, triplet, quartet, and multiplet, respectively; rt denotes room temperature.

The purity of all compounds evaluated in the biological assays was >95%, as determined by HPLC/MS. EI mass spectra were recorded on a Shimadzu QP5050 GC/MS instrument at 75 eV. Elemental analyses were within ±0.4% of the calculated values. TLC was carried out on precoated silica gel GF254 plates (0.25 mm) and compounds were visualized under UV light at 254 nm. Column chromatography was performed using Merck silica gel (230 or 400 mesh) or a Biotage FLASH40i system equipped with prepacked cartridges. General analytical and chromatographic procedures were performed according to previously reported protocols.^38–41^

2-Chloro-6-Hydroxy-pyridine (**8**), benzoyl chlorides **9a**–**k** and benzoic acids **9l**,**m** were commercially available. The final products **7a**–**m** were prepared through two alternative synthetic routes, as outlined in Scheme 1.

#### General Procedure for the Synthesis of 6-Chloropyridin-2-yl Benzoate **7a**–**m**

*Route A*: 2-Chloro-6-hydroxypyridine (**8**, 1.0 equiv) was dissolved in pyridine (1.2 equiv) and cooled to 0 °C. A solution of the appropriate benzoyl chloride **9a**–**k** (1.2 equiv) in DCM was then added, and the reaction mixture was allowed to reach room temperature and stirred for 12–24 h. Upon completion, the solvent was removed under reduced pressure, and the residue was poured into an ice/water mixture. The aqueous phase was extracted with DCM, and the combined organic layers were washed with 1 M HCl, dried over Na_2_SO_4_, and concentrated. The crude material was purified by silica gel column chromatography using petroleum ether/EtOAc (10:1, gradient elution) to give compounds **7a**–**k** as white powders.

R*oute B*: A mixture of 2-chloro-6-hydroxypyridine (**8**, 1.0 equiv), the appropriate benzoic acid **9l**,**m** (1.0 equiv), and DCC (1.5 equiv) in anhydrous DCM was stirred under a nitrogen atmosphere. After cooling the mixture to 0 °C, DMAP (0.5 equiv) was added, and the reaction was subsequently maintained at room temperature for 12–24 h. The solvent was then removed under reduced pressure, and the residue was treated with an ice/water mixture and extracted with DCM. The organic phase was dried over Na_2_SO_4_ and concentrated, and purification by silica gel chromatography using petroleum ether/EtOAc (10:1, gradient elution) afforded compounds **7l**,**m** as white powders.

##### 6-Chloropyridin-2-yl Benzoate (**7a**)

Yield 81%. Mp 48–50 °C. ^1^H NMR (DMSO-*d*_*6*_) δ: 7.44 (dd, 1H, *J* = 8.0, 0.6 Hz, H-3), 7.58 (dd, 1H, *J* = 7.8, 0.6 Hz, H-5), 7.60–7.66 (m, 2H, H-3′, H-5′), 7.75–7.82 (m, 1H, H-4′), 8.09 (t, 1H, *J* = 7.9 Hz, H-4), 8.12–8.16 (m, 2H, H-2′, H-6′). ^13^C NMR (DMSO-*d*_*6*_) δ: 116.5 (CH), 123.5 (CH), 128.5, 129.6 (CH), 130.4 (CH), 135.0 (CH), 143.8 (CH), 148.6, 157.4, 164.5. HRMS-ESI [(M+H)^+^]: *m*/*z* calculated for C_12_H_8_ClNO_2_: 234.0316; found: 234.0314.

##### 6-Chloropyridin-2-yl 4-methylbenzoate (**7b**)

Yield 85%. Mp 45–50 °C. ^1^H NMR (DMSO-*d*_*6*_) δ: 2.43 (s, 3H, CH_3_), 7.39–7.47 (m, 3H, H-3, H-3′, H-5′), 7.56 (d, 1H, *J* = 7.8 Hz, H-5), 8.02 (d, 2H, *J* = 8.2 Hz, H-2′, H-6′), 8.07 (t, 1H, *J* = 7.9 Hz, H-4). ^13^C NMR (DMSO-*d*_*6*_) δ: 21.8 (CH_3_), 116.5 (CH), 123.4 (CH), 125.7, 130.2 (CH), 130.5 (CH), 143.8 (CH), 145.7, 148.6, 157.5, 164.4. HRMS-ESI [(M+H)^+^]: *m*/*z* calculated for C_13_H_10_ClNO_2_: 248.0473; found: 248.0475.

##### 6-Chloropyridin-2-yl 4-methoxybenzoate (**7c**)

Yield 83%. Mp 88 °C. ^1^H NMR (DMSO-*d*_*6*_) δ: 3.88 (s, 3H, OCH_3_), 7.14 (d, 2H, *J* = 9.0 Hz, H-3′, H-5′), 7.40 (dd, 1H, *J* = 8.0, 0.6 Hz, H-3), 7.55 (dd, 1H, *J* = 7.8, 0.6 Hz, H-5), 8.02–8.13 (m, 3H, H-4, H-2′, H-6′). ^13^C NMR (DMSO-*d*_*6*_) δ: 56.2 (CH_3_), 114.9 (CH), 116.5 (CH), 120.5, 123.3 (CH), 132.7 (CH), 143.7 (CH), 148.5, 157.6, 164.1, 164.7. HRMS-ESI [(M+H)^+^]: *m*/*z* calculated for C_13_H_10_ClNO_3_: 264.0422; found: 264.0420.

##### 6-Chloropyridin-2-yl 4-(trifluoromethyl)­benzoate (**7d**)

Yield 89%. Mp 58–60 °C. ^1^H NMR (DMSO-*d*_*6*_) δ: 7.49 (dd, 1H, *J* = 7.9, 0.6 Hz, H-3), 7.60 (dd, 1H, *J* = 7.8, 0.6 Hz, H-5), 8.01 (d, 2H, *J* = 8.3 Hz, H-3′, H-5′), 8.11 (t, 1H, *J* = 7.9 Hz, H-4), 8.33 (d, 2H, *J* = 8.1 Hz, H-2′, H-6′). ^13^C NMR (DMSO-*d*_*6*_) δ: 116.4 (CH), 123.8 (CH), 124.1, 126.6 (CH), 131.3 (CH), 132.4, 134.3, 143.9 (CH), 148.6, 157.1, 163.4. HRMS-ESI [(M+H)^+^]: *m*/*z* calculated for C_13_H_7_ClF_3_NO_2_: 302.0190; found: 302.0190.

##### 6-Chloropyridin-2-yl 4-(dimethylamino)­benzoate (**7e**)

Yield 91%. Mp 115–118 °C. ^1^H NMR (DMSO-*d*_*6*_) δ: 3.05 (s, 6H, 2 x CH_3_), 6.80 (d, 2H, *J* = 9.1 Hz, H-3′, H-5′), 7.34 (dd, 1H, *J* = 8.0, 0.6 Hz, H-3), 7.51 (dd, 1H, *J* = 7.8, 0.6 Hz, H-5), 7.91 (d, 2H, *J* = 9.0 Hz, H-2′, H-6′), 8.03 (t, 1H, *J* = 7.9 Hz, H-4). ^13^C NMR (DMSO-*d*_*6*_) δ: 40.1 (CH_3_), 111.5 (CH), 113.8, 116.6 (CH), 122.8 (CH), 132.3 (CH), 143.4 (CH), 148.5, 154.5, 158.0, 164.4. HRMS-ESI [(M+H)^+^]: *m*/*z* calculated for C_14_H_13_ClN_2_O_2_: 277.0738; found: 277.0740.

##### 6-Chloropyridin-2-yl 4-fluorobenzoate (**7f**)

Yield 92%. Mp 70–71 °C. ^1^H NMR (DMSO-*d*_*6*_) δ: 7.41–7.50 (m, 3H, H-3, H-3′, H-5′), 7.58 (d, 1H, *J* = 7.8 Hz, H-5), 8.09 (t, 1H, *J* = 7.9 Hz, H-4), 8.17–8.25 (m, 2H, H-2′, H-6′). ^13^C NMR (DMSO-*d*_*6*_) δ: 116.5 (CH), 116.8 (d, J_C–F_ = 22.3 Hz, CH), 123.5 (CH), 125.1, 133.5 (d, J_C–F_ = 9.8 Hz, CH), 143.8 (CH), 148.6, 157.3, 163.5, 166.3 (d, *J* = 253.8 Hz). HRMS-ESI [(M+H)^+^]: *m*/*z* calculated for C_12_H_7_ClFNO_2_: 252.0222; found: 252.0220.

##### 6-Chloropyridin-2-yl 4-bromobenzoate (**7g**)

Yield 89%. Mp 129–133 °C. ^1^H NMR (DMSO-*d*_*6*_) δ: 7.45 (dd, 1H, *J* = 8.0, 0.6 Hz, H-3), 7.58 (dd, 1H, *J* = 7.8, 0.6 Hz, H-5), 7.84 (d, 2H, *J* = 8.6 Hz, H-3′, H-5′), 8.05 (d, 2H, *J* = 8.6 Hz, H-2′, H-6′), 8.09 (t, 1H, *J* = 7.9 Hz, H-4). ^13^C NMR (DMSO-*d*_*6*_) δ: 116.5 (CH), 123.6 (CH), 127.8, 129.3, 132.3 (CH), 132.8 (CH), 143.9 (CH), 148.6, 157.2, 163.8. HRMS-ESI [(M+H)^+^]: *m*/*z* calculated for C_12_H_7_BrClNO_2_: 311.9421; found: 311.9420.

##### 6-Chloropyridin-2-yl 4-chlorobenzoate (**7h**)

Yield 90%. Mp 117–120 °C. ^1^H NMR (DMSO-*d*_*6*_) δ: 7.45 (dd, 1H, *J* = 8.0, 0.7 Hz, H-3), 7.58 (dd, 1H, *J* = 7.8, 0.6 Hz, H-5), 7.70 (d, 2H, *J* = 8.7 Hz, H-3′, H-5′), 8.09 (t, 1H, *J* = 7.9 Hz, H-4), 8.13 (d, 2H, *J* = 8.7 Hz, H-2′, H-6′). ^13^C NMR (DMSO-*d*_*6*_) δ: 116.5 (CH), 123.6 (CH), 127.4, 129.8 (CH), 132.3 (CH), 140.1, 143.9 (CH), 148.6, 157.2, 163.6. HRMS-ESI [(M+H)^+^]: *m*/*z* calculated for C_12_H_7_Cl_2_NO_2_: 267.9927; found: 267.9927.

##### 6-Chloropyridin-2-yl 3,4,5-trimethoxybenzoate (**7i**)

Yield 85%. Mp 142–145 °C. ^1^H NMR (DMSO-*d*_*6*_) δ: 3.79 (s, 3H, OCH_3_), 3.87 (s, 6H, 2 x OCH_3_), 7.38–7.44 (m, 3H, H-3, H-2′, H-6′), 7.57 (d, 1H, *J* = 7.7 Hz, H-5), 8.08 (t, 1H, *J* = 7.9 Hz, H-4). ^13^C NMR (DMSO-*d*_*6*_) δ: 56.7 (CH_3_), 60.8 (CH_3_), 107.8 (CH), 116.5 (CH), 123.3, 123.5 (CH), 143.3, 143.8 (CH), 148.6, 153.4, 157.4, 164.1. HRMS-ESI [(M+H)^+^]: *m*/*z* calculated for C_15_H_14_ClNO_5_: 324.0633; found: 324.0635.

##### 6-Chloropyridin-2-yl 2,3-dichlorobenzoate (**7j**)

Yield 80%. Mp 98–100 °C. ^1^H NMR (DMSO-*d*_*6*_) δ: 7.49 (dd, 1H, *J* = 8.0, 0.6 Hz, H-3), 7.55–7.64 (m, 2H, H-5, H-5′), 7.98 (dd, 1H, *J* = 8.1, 1.5 Hz, H-6′), 8.08 (dd, 1H, *J* = 7.8, 1.6 Hz, H-4′), 8.12 (t, 1H, *J* = 7.9 Hz, H-4). ^13^C NMR (DMSO-*d*_*6*_) δ: 116.3 (CH), 123.9 (CH), 129.2 (CH), 130.8, 130.9 (CH), 131.2, 134.1, 135.2 (CH), 144.0 (CH), 148.7, 156.8, 162.6. HRMS-ESI [(M+H)^+^]: *m*/*z* calculated for C_12_H_6_Cl_3_NO_2_: 301.9537; found: 301.9535.

##### 6-Chloropyridin-2-yl 3-chloro-4-fluorobenzoate (**7k**)

Yield 94%. Mp 90–92 °C. ^1^H NMR (DMSO-*d*_*6*_) δ: 7.45 (dd, 1H, *J* = 8.0, 0.6 Hz, H-3), 7.59 (dd, 1H, *J* = 7.8, 0.6 Hz, H-5), 7.68 (t, 1H, *J* = 8.9 Hz, H-5′), 8.10 (t, 1H, *J* = 7.9 Hz, H-4), 8.16 (ddd, 1H, *J* = 8.7, 4.7, 2.2 Hz, H-6′), 8.29 (dd, 1H, *J* = 7.1, 2.2 Hz, H-2′). ^13^C NMR (DMSO-*d*_*6*_) δ: 116.4 (CH), 118.3 (d, J_C–F_ = 22.1 Hz, CH), 121.2 (d, J_C–F_ = 18.4 Hz), 123.7 (CH), 126.4 (d, J_C–F_ = 3.3 Hz), 131.9 (d, J_C–F_ = 9.0 Hz, CH), 132.9 (CH), 143.9 (CH), 148.6, 157.0, 161.4 (d, J_C–F_ = 256 Hz), 162.5. HRMS-ESI [(M+H)^+^]: *m*/*z* calculated for C_12_H_6_Cl_2_FNO_2_: 285.9832; found: 285.9830.

##### 6-Chloropyridin-2-yl 5-chloro-2-nitrobenzoate (**7l**)

Yield 87%. ^1^H NMR (DMSO-*d*_*6*_) δ: 7.46 (dd, 1H, *J* = 8.0, 0.6 Hz, H-3), 7.63 (dd,1 H, *J* = 7.8, 0.6 Hz, H-5), 8.03 (dd, 1H, *J* = 8.8, 2.3 Hz, H-4′), 8.14 (t, 1H, *J* = 7.9 Hz, H-4), 8.26 (d, 1H, *J* = 8.8 Hz, H-3′), 8.29 (d, 1H, *J* = 2.3 Hz, H-6). ^13^C NMR (DMSO-*d*_*6*_) δ: 115.9, 124.3, 127.1, 127.3, 130.6, 133.9, 139.4, 144.2, 146.3, 148.7, 156.2, 162.0. HRMS-ESI [(M+H)^+^]: *m*/*z* calculated for C_12_H_6_Cl_2_N_2_O_4_: 312.9777; found: 312.9778.

##### 6-Chloropyridin-2-yl 2-amino-3,5-dichlorobenzoate (**7m**)

Yield 82%. ^1^H NMR (DMSO-*d*_*6*_) δ: 6.95 (br s, 2H, NH_2_), 7.43 (dd, 1H, *J* = 8.0, 0.7 Hz, H-3), 7.58 (dd, 1H, *J* = 7.8, 0.6 Hz, H-5), 7.80 (d, 1H, *J* = 2.5 Hz, H-4′), 7.93 (d, 1H, *J* = 2.5 Hz, H-6′), 8.09 (t, 1H, *J* = 7.9 Hz, H-4). ^13^C NMR (DMSO-*d*_*6*_) δ: 109.7, 116.6 (CH), 118.5, 121.0, 123.6 (CH), 125.9, 129.7 (CH), 135.0 (CH), 143.8 (CH), 147.3, 148.5, 164.4. HRMS-ESI [(M+H)^+^]: *m*/*z* calculated for C_12_H_7_Cl_3_N_2_O_2_: 316.9646; found: 316.9647.

### Biology

#### Cell Culture and Treatment

Human melanoma A-375, colorectal carcinoma HCT-116, breast cancer MCF-7 and nontumorigenic bronchial epithelial 16-HBE cells were obtained from ATCC (Rockville, MD). Cells were grown in 75 cm^2^ flasks in DMEM containing 10% FBS, 1% nonessential amino acids, 10 mM HEPES, 50 U/mL penicillin and 50 μg/mL streptomycin, at 37 °C in a humidified 5% CO_2_ atmosphere. Cultures were passaged twice a week at 5.5 × 10^4^ cells/cm^2^ to keep them in exponential growth, and were used between passages 3 and 10. Compounds were prepared as DMSO stocks and diluted in medium to a final DMSO concentration of 0.1% or lower. Cells were plated in 24- or 96-well plates, allowed to attach overnight and exposed for 24 h to the compounds or to vehicle. Vehicle alone (0.1% DMSO) did not change cell number or viability compared with untreated cells.

#### Cell Viability

Viability was measured with the MTT assay.^42^ Cells were plated in 96-well plates (Corning Costar, Milan, Italy) at 5.5 × 10^4^ cells/cm^2^ in 200 μL DMEM. After overnight attachment, the medium was renewed and cells were exposed to the compounds (0.1–100 μM) for 24 h. The medium was then replaced with 200 μL of serum-free DMEM containing 4 μL of MTT (5 mg/mL). After 2 h at 37 °C, plates were centrifuged and the medium removed. The formazan produced by viable cells was dissolved in 100 μL DMSO and absorbance was read at 570 nm (LTek INNO microplate reader, Seongnam, Republic of Korea). Viability was expressed as a percentage of vehicle-treated controls. IC_50_ values were calculated with a dose–response inhibition model in GraphPad Prism 9.5.0 (San Diego, CA). The selectivity index was the ratio between the IC_50_ in 16-HBE cells and the IC_50_ in A-375 cells.^43^ Each condition was tested in triplicate in [*n*] independent experiments.

#### Measurement of Phosphatidylserine Exposure

Phosphatidylserine exposure was measured with the Annexin V-FITC Apoptosis Detection Kit (cat. 88-8005-74, eBioscience, San Diego, CA) following the manufacturer’s protocol.^44^ A-375 cells were plated in triplicate in 24-well plates (5.5 × 10^4^ cells/cm^2^), left to attach overnight and treated for 24 h as described above. Cells were then detached with trypsin, collected and resuspended in the kit binding buffer at 1.0 × 10^6^ cells/mL. Aliquots of 100 μL were stained with 5 μL Annexin V-FITC and 10 μL PI (20 μg/mL) for 20 min at room temperature in the dark. At least 1.0 × 10^4^ events per sample were acquired on a CytoFLEX B2-R2-V0 flow cytometer and analyzed with CytExpert software (Beckman Coulter, Brea, CA).

#### Measurement of Mitochondrial Membrane Potential

ΔΨm was assessed with DiOC6 (Molecular Probes, Life Technologies Italia, Monza, Italy). This lipophilic cationic dye accumulates in the matrix of polarized mitochondria.^45^ After 24 h of treatment, cells were loaded with 40 nM DiOC6 for 15 min at 37 °C in the dark. They were then washed, pelleted and resuspended in 500 μL PBS. Green fluorescence was recorded on at least 1.0 × 10^4^ cells per sample. A reduction in DiOC6 signal was taken as loss of ΔΨm.

#### Measurement of Intracellular Reactive Oxygen Species

Intracellular ROS were measured with 2′,7′-dichlorodihydrofluorescein diacetate (DCFDA; Merck, Milan, Italy).^46^ The probe (10 μM) was added to the medium during the last 30 min of treatment. Cells were then washed, detached with trypsin, pelleted (2000 rpm, 5 min, 4 °C) and resuspended in PBS. The fluorescence of oxidized DCF was recorded on at least 1.0 × 10^4^ cells per sample.

#### Cell Cycle Analysis

After 24 h of treatment, cells were detached with trypsin and pelleted. Aliquots of 1.0 × 10^6^ cells were washed in PBS and stained for 30 min at room temperature in the dark with PBS containing 0.1% (v/v) Triton X-100, 20 μg/mL PI and 200 μg/mL RNase.^47^ Samples were acquired immediately, recording at least 1.0 × 10^4^ events each. The fractions of cells in G0/G1, S and G2/M were derived from the DNA content histograms.

#### Statistical Analysis

Results are shown as mean ± SD of n independent experiments, each run in triplicate. The value of n is given in each figure legend. Groups were compared by one-way ANOVA followed by Tukey’s post hoc test in GraphPad Prism 9.5.0. Differences with *p* < 0.05 were considered significant.

### In Silico Studies

#### In Silico ADME and Drug-Likeness Assessment

Physicochemical, drug-likeness, and ADME-related properties of the designed compounds were initially evaluated using the SwissADME web-based platform.^16,18^ The SMILES representation of each compound was submitted to the server to obtain relevant physicochemical and ADME-related descriptors, together with the BOILED-Egg and bioavailability radar plots. QikProp (Release 2017-1; Schrödinger LLC, New York, NY), implemented in the Schrödinger Maestro Suite (Maestro version 11.1.012), was subsequently employed to further characterize the predicted physicochemical and pharmacokinetic profiles of the compounds.^17^ The molecular structures were generated as SDF files and prepared using the LigPrep workflow, including energy minimization, prior to QikProp calculations. All predictions were performed using the standard protocol provided with the software package. The complementary application of SwissADME and QikProp enabled a broader assessment of the predicted absorption, distribution, physicochemical, and overall drug-likeness profiles of the investigated compounds.

#### Density Functional Theory (DFT) Analysis

Density Functional Theory (DFT) calculations were performed for the selected compounds using the Jaguar module implemented in the Schrödinger Maestro Suite (Release 2017-1; Schrödinger LLC, New York, NY).^37^ The molecular geometries were fully optimized at the DFT level using the B3LYP hybrid functional in combination with the 6–31G** basis set. The B3LYP functional was selected because it represents a well-established and widely used approach for the description of the electronic structure of organic molecules, providing a reliable balance between computational cost and accuracy for the calculation of molecular geometries and frontier molecular orbital energies. All calculations were carried out assuming neutral molecules with singlet spin multiplicity.

Geometry optimizations were performed using the default convergence criteria implemented in Jaguar, while the SCF procedure employed the DIIS convergence scheme. Solvent effects were taken into account using the Poisson–Boltzmann Finite (PBF) implicit solvation model with water as the solvent (dielectric constant ε = 80.37).

Following geometry optimization, frontier molecular orbital (FMO) analysis and electrostatic potential calculations were performed. The energies of the highest occupied molecular orbital (HOMO) and lowest unoccupied molecular orbital (LUMO), expressed in Hartree, were used to estimate global reactivity descriptors within the framework of Koopmans’ approximation. Accordingly, the ionization potential (IP) and electron affinity (EA) were obtained from the negative values of the HOMO and LUMO energies, respectively. Based on these quantities, additional descriptors were calculated, including chemical hardness (η), defined as (IP – EA)/2; chemical potential (μ), defined as −(IP + EA)/2; electronegativity (χ), defined as (IP + EA)/2; global softness (S), calculated as 1/(2η); and the electrophilicity index (ω), defined as μ^2^/(2η). These parameters were used to evaluate the electronic reactivity of the selected compounds and to support the discussion of their biological activity.

## Supplementary Material





## Notes

Drug-likeness bioavailability radar plot provided by SwissADME for compounds **7a**–**m** (Figure S1); and ^1^HNMR and ^13^CNMR spectra for compounds **7a**–**m** (Figures S2–27) (PDF)
QikProp output matrix for compounds **7a**–**m** (Table S1); SwissADME output matrix for compounds **7a**–**m** (Table S2) (XLSX)

## Abbreviations

ADME: absorption distribution metabolism and excretion
ANOVA: analysis of variance
BBB: blood–brain barrier
DCC: dicyclohexylcarbodiimide
DCFDA: 2′,7′-dichlorodihydrofluorescein diacetate
DCM: dichloromethane
DFT: density functional theory
DiOC6: 3,3′-dihexyloxacarbocyanine iodide
DMAP: 4-dimethylaminopyridine
DMEM: Dulbecco’s Modified Eagle Medium
DMSO: dimethyl sulfoxide
EDG: electron-donating group
EWG: electron-withdrawing group
FBDD: fragment-based drug discovery
FBS: fetal bovine serum
FITC: fluorescein isothiocyanate
GI: gastrointestinal
HOMO: highest occupied molecular orbital
HRMS: high-resolution mass spectrometry
IC_50_: half-maximal inhibitory concentration
LUMO: lowest unoccupied molecular orbital
MEP: molecular electrostatic potential
MMP: mitochondrial membrane potential
MTT: 3-(4,5-dimethylthiazol-2-yl)-2,5-diphenyltetrazolium bromide
PDD: phenotypic drug discovery
PI: propidium iodide
ROS: reactive oxygen species
SAR: structure–activity relationship
SD: standard deviation
SI: selectivity index
TPSA: topological polar surface area
ΔΨm: mitochondrial membrane potential
