# Supplementary material for: 6‑Chloropyridinyl Benzoate Fragments as Selective Anti-Melanoma Scaffolds Triggering ROS-Mediated Mitochondrial Apoptosis
Source: ACS Omega. 2026 Oct 12;11(42):65020–34. doi: 10.1021/acsomega.6c06714 (PMC13717780; doi:10.1021/acsomega.6c06714)

Supporting Information

6-Chloropyridinyl Benzoate Fragments as Selective Anti-Melanoma Scaffolds Triggering ROS-Mediated Mitochondrial Apoptosis

Gabriele La Monica,[a,‡] Ignazio Restivo,[a,‡] Alessia Bono,[a,b] Federica Alamia,[a] Dennis Tocco,[a] Alessandro Attanzio,[a,] Antonino Lauria[a] and Annamaria Martorana[a,*]*

[a] Dipartimento di Scienze e Tecnologie Biologiche Chimiche e Farmaceutiche "STEBICEF", University of Palermo, 90123 Palermo, Italy.

[b] Fondazione Umberto Veronesi (FUV), via Solferino 19, Milano 20121, Italy.

‡Co-first authorship

**Corresponding Authors;*

e-mail addresses: annamaria.martorana@unipa.it; alessandro.attanzio@unipa.it

Table of contents

Supporting Tables

- **Table S1:** QikProp output matrix for compounds **7a–m** (attached .xlsx file).
- **Table S2:** SwissADME output matrix for compounds **7a–m** (attached .xlsx file).

Supporting Figures

- **Figure S1:** Drug-likeness bioavailability radar plot provided by SwissADME for compounds **7a–m**.
- **Figures S2–27:** ^{1}HNMR and ^{13}CNMR spectra for compounds **7a–m**:
    - *S2-3*: ^{1}HNMR and ^{13}CNMR for compound **7a** (d_6-DMSO).
    - *S4-5*: ^{1}HNMR and ^{13}CNMR for compound **7b** (d_6-DMSO).
    - *S6-7*: ^{1}HNMR and ^{13}CNMR for compound **7c** (d_6-DMSO).
    - *S8-9*: ^{1}HNMR and ^{13}CNMR for compound **7d** (d_6-DMSO).
    - *S10-11:* ^{1}HNMR and ^{13}CNMR for compound **7e** (d_6-DMSO).
    - *S12-13*: ^{1}HNMR and ^{13}CNMR for compound **7f** (d_6-DMSO).
    - *S14-15*: ^{1}HNMR and ^{13}CNMR for compound **7g** (d_6-DMSO).
    - *S16-17*: ^{1}HNMR and ^{13}CNMR for compound **7h** (d_6-DMSO).
    - *S18-19*: ^{1}HNMR and ^{13}CNMR for compound **7i** (d_6-DMSO).
    - *S20-21*: ^{1}HNMR and ^{13}CNMR for compound **7j** (d_6-DMSO).
    - *S22-23*: ^{1}HNMR and ^{13}CNMR for compound **7k** (d_6-DMSO).
    - *S24-25*: ^{1}HNMR and ^{13}CNMR for compound **7l** (d_6-DMSO).
    - *S26-27*: ^{1}HNMR and ^{13}CNMR for compound **7m** (d_6-DMSO).

Figure S1: Drug-likeness bioavailability radar plot provided by SwissADME for compounds **7a–m**.

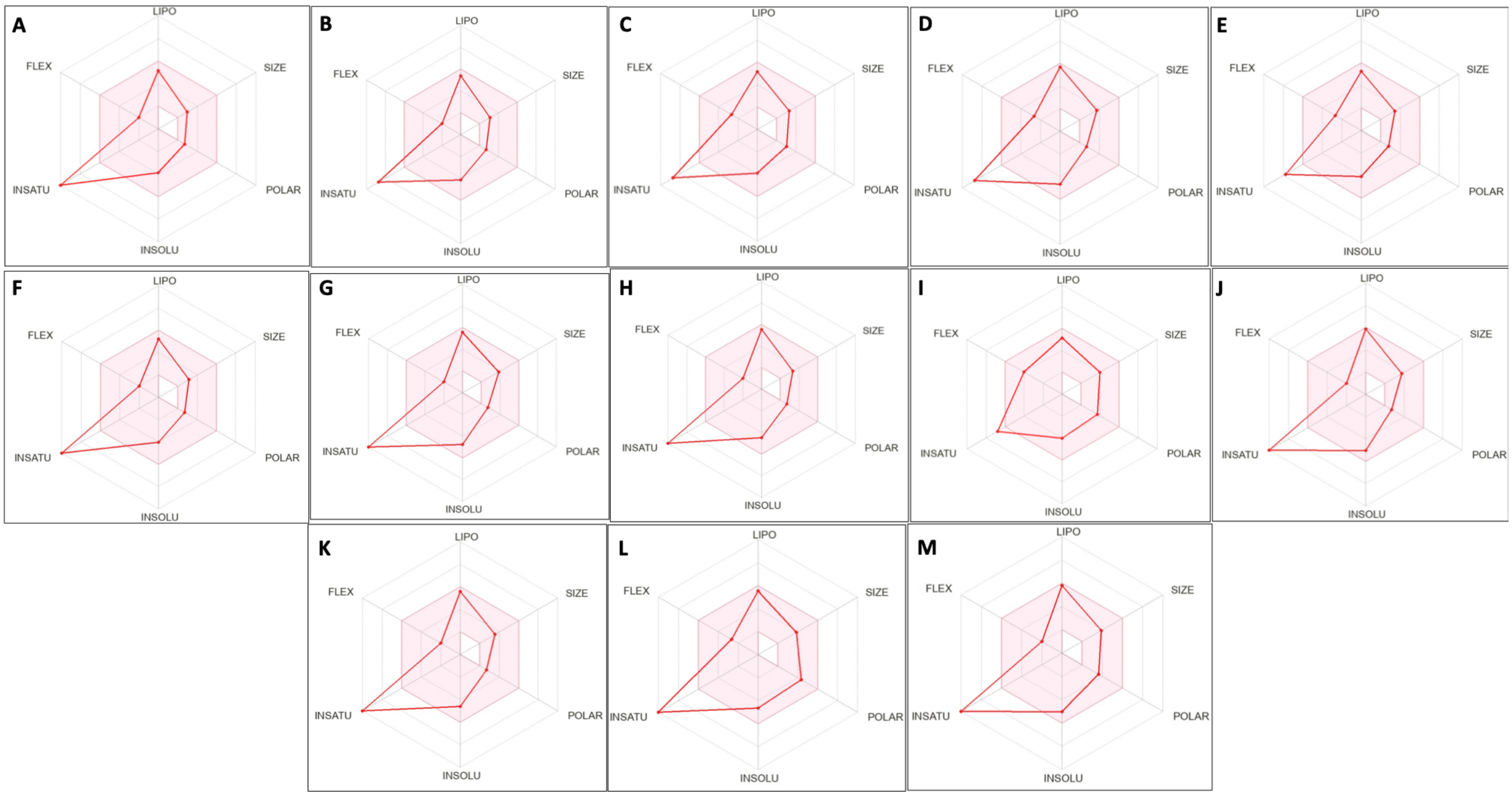

Bioavailability radar plots of the synthesized compounds predicted using SwissADME. The colored region represents the optimal physicochemical space for oral bioavailability, defined by the following parameters: lipophilicity (−0.7 < XLOGP3 < +5.0), molecular weight (150 < MW < 500 g/mol), polarity (20 Å^2 < TPSA < 130 Å^2), solubility (−6 < logS (ESOL) < 0), saturation (0.25 < fraction Csp^3 < 1), and flexibility (number of rotatable bonds < 9).

S2: ^{1}HNMR compound **7a** (DMSO-d$_6$)

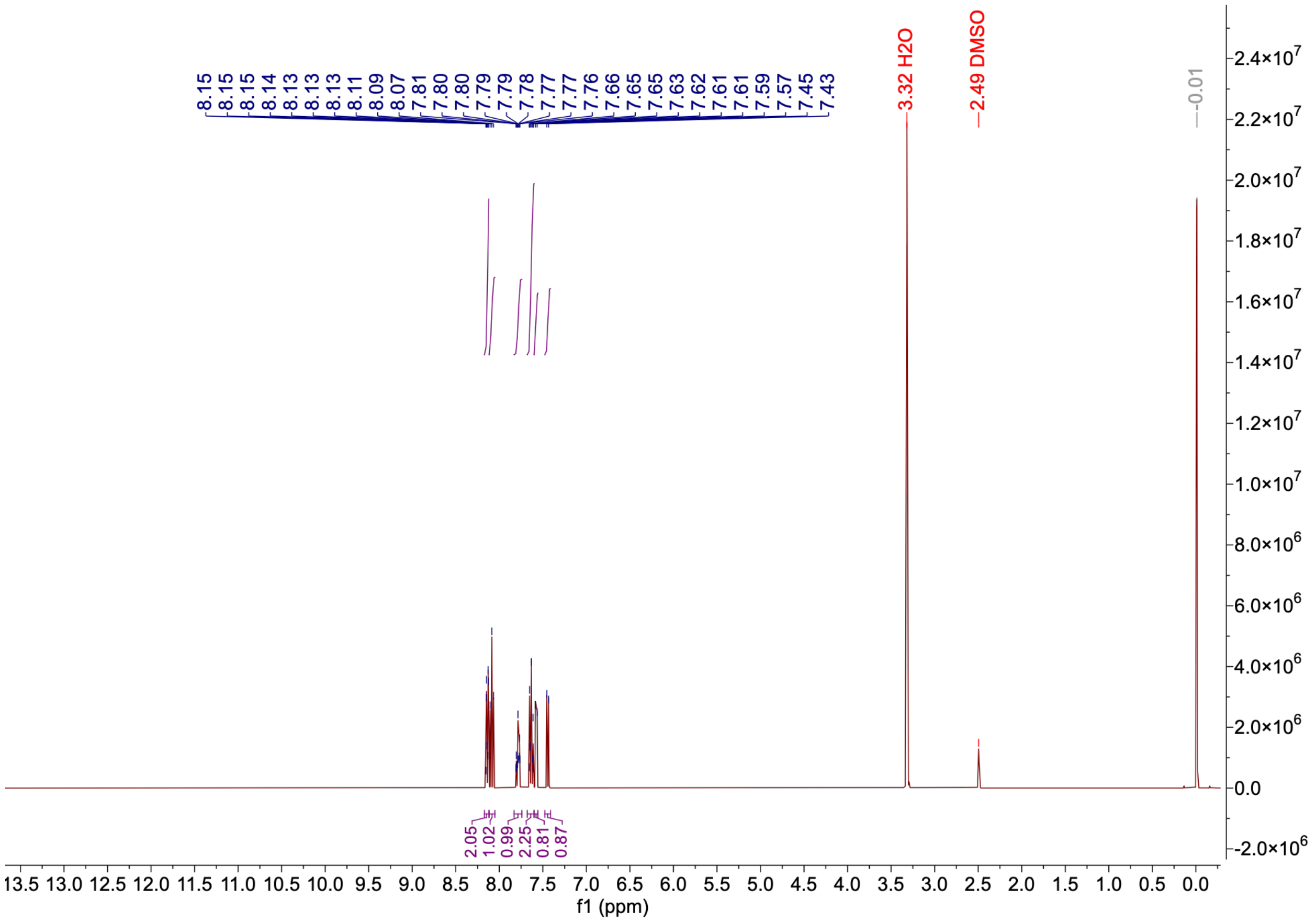

S3: ^{13}CNMR compound **7a** (DMSO-d$_6$)

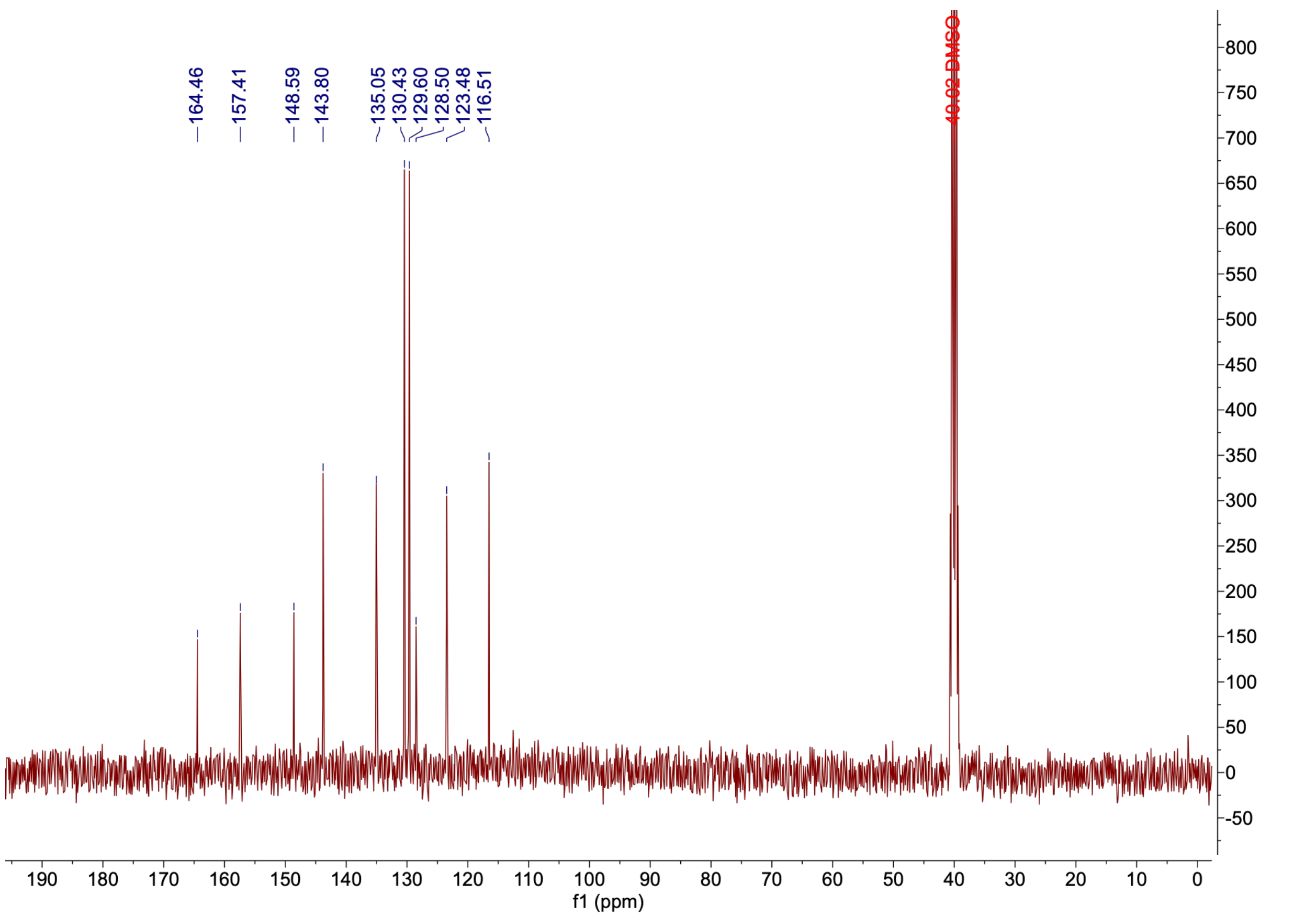

S4: ^{1}HNMR compound **7b** (DMSO-d$_6$)

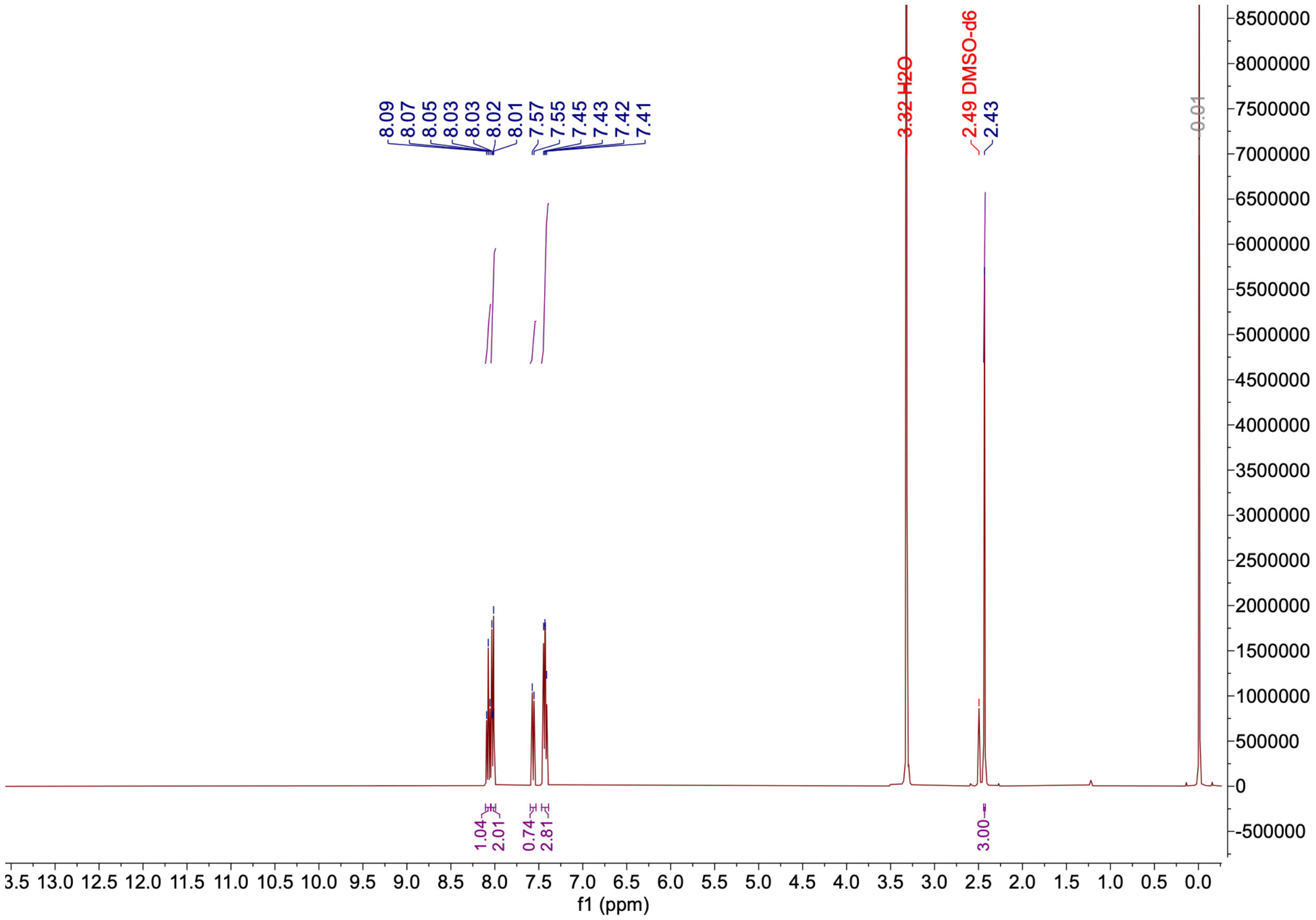

S5: ^{13}CNMR compound **7b** (DMSO-d$_6$)

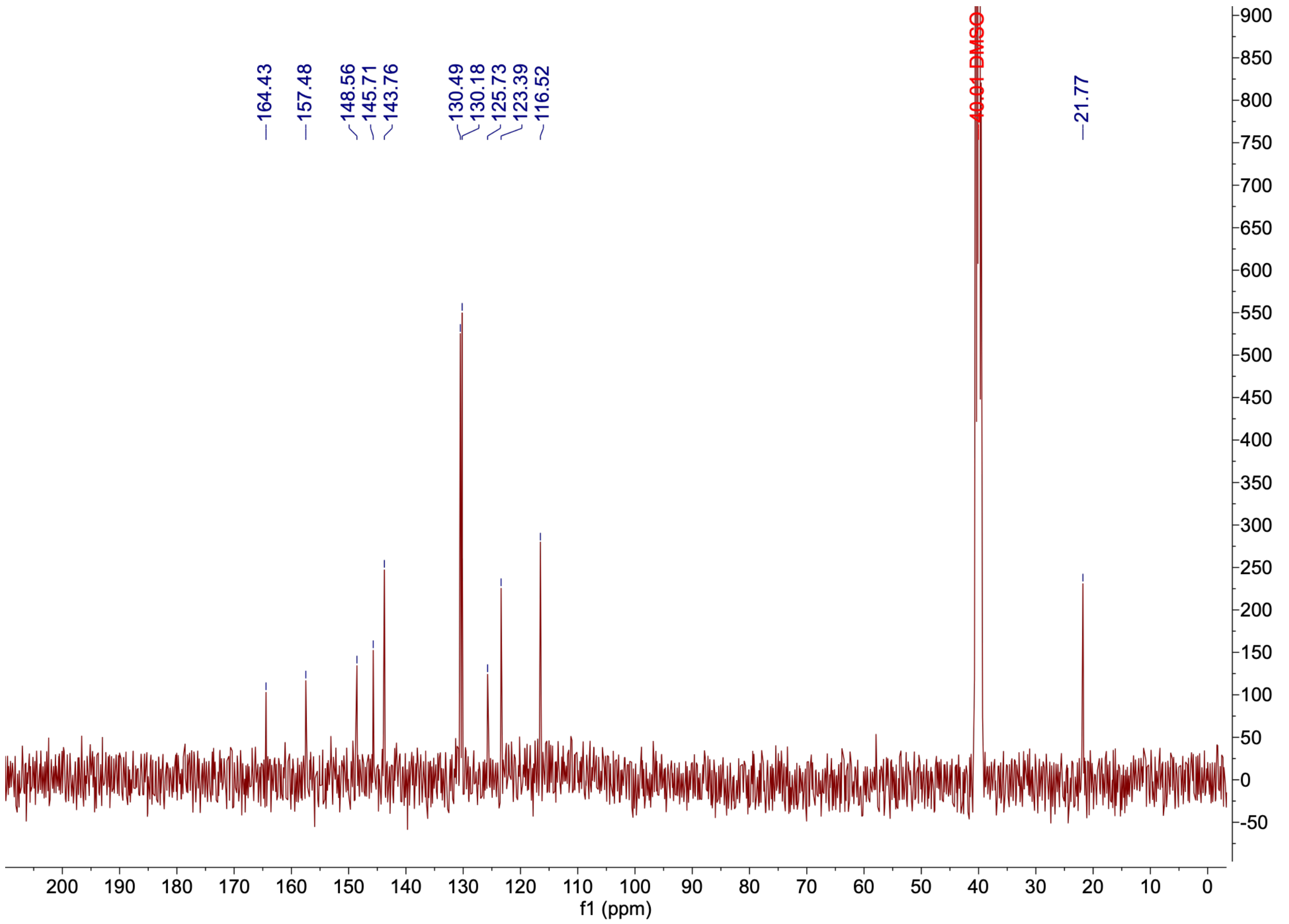

S6: ^{1}HNMR compound **7c** (DMSO-d_6)

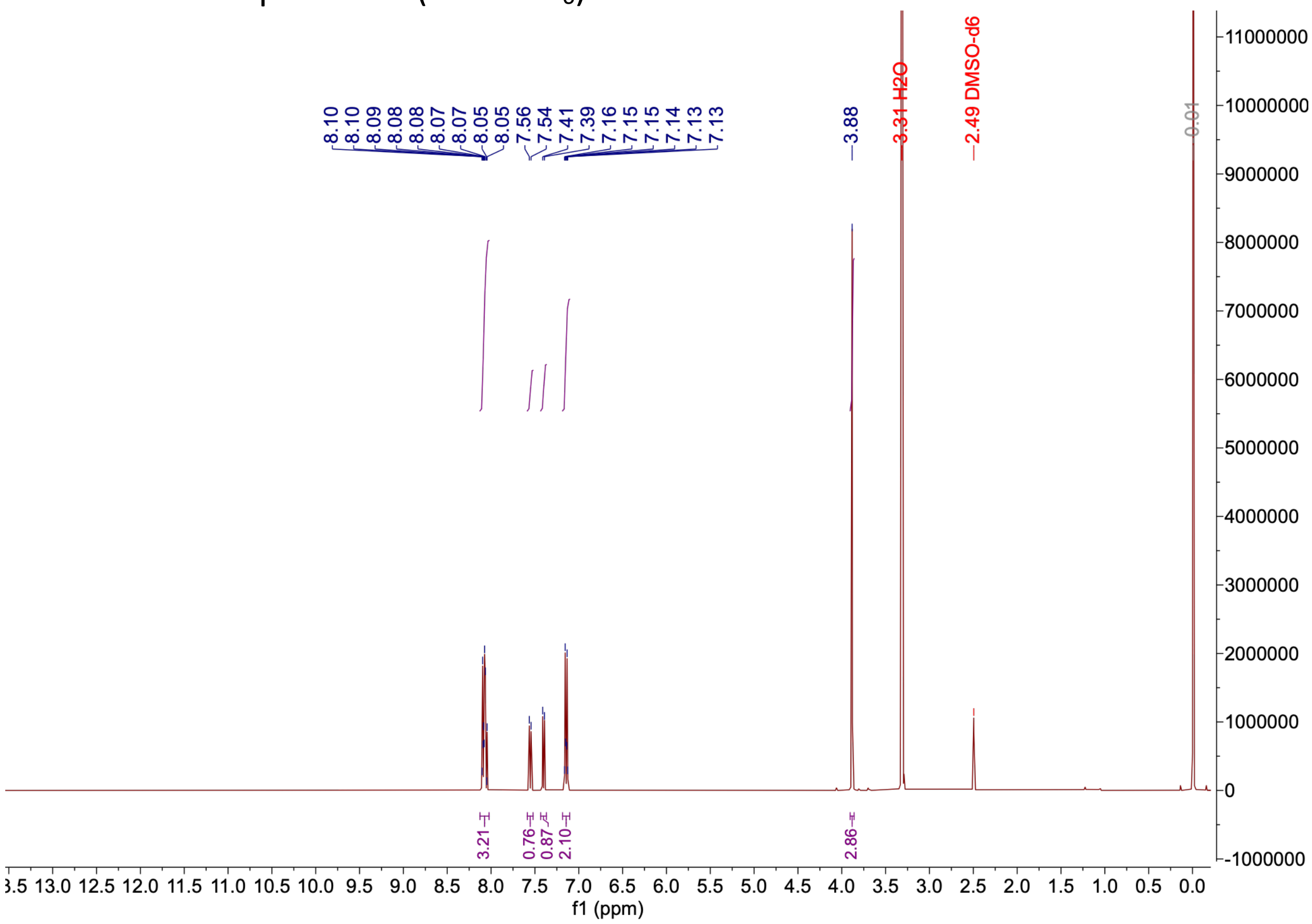

S7: ^{13}CNMR compound **7c** (DMSO-d_6)

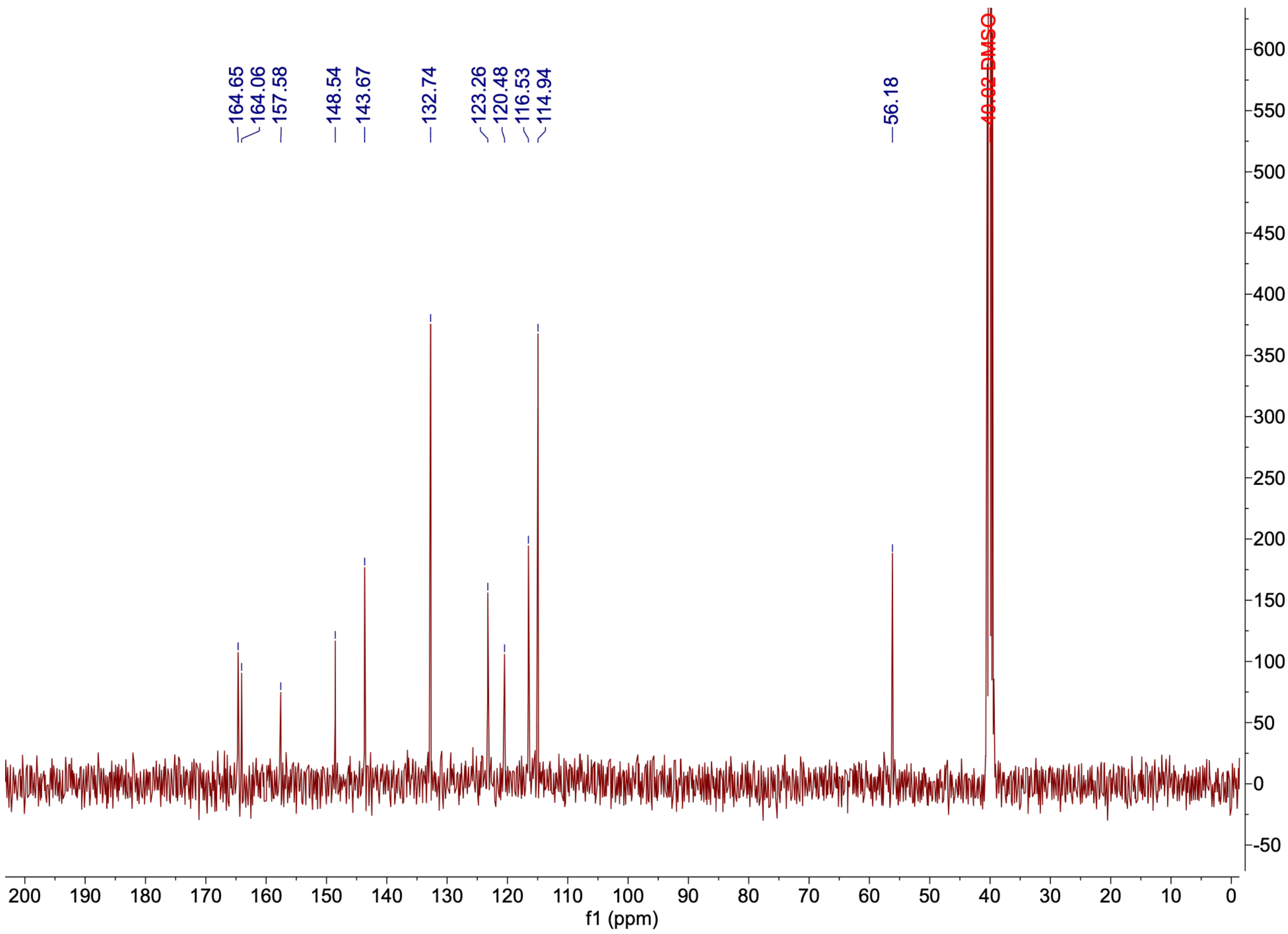

S8: ^{1}HNMR compound **7d** (DMSO-d_6)

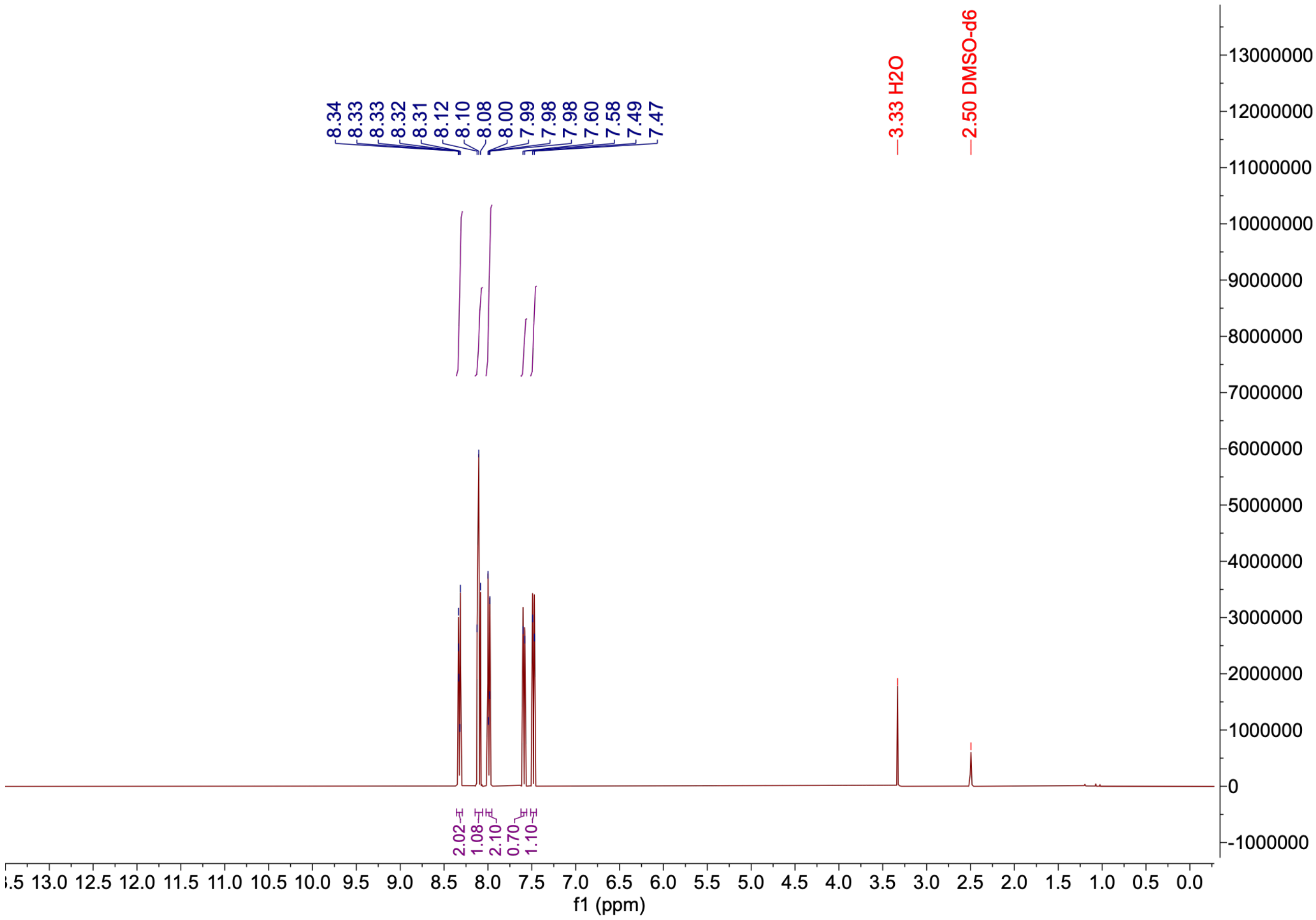

S9: ^{13}CNMR compound **7d** (DMSO-d_6)

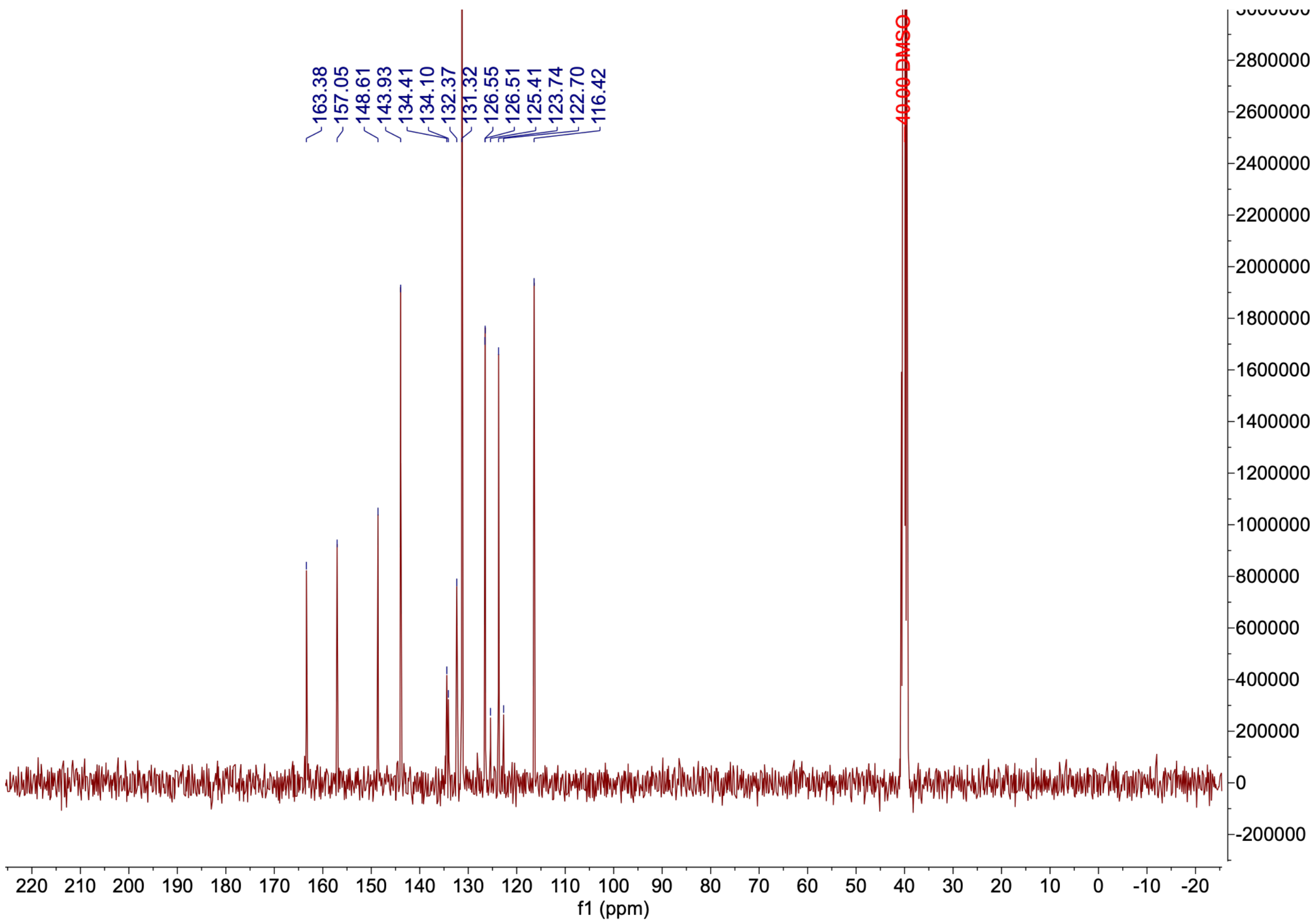

S10: ^{1}HNMR compound **7e** (DMSO-d_6)

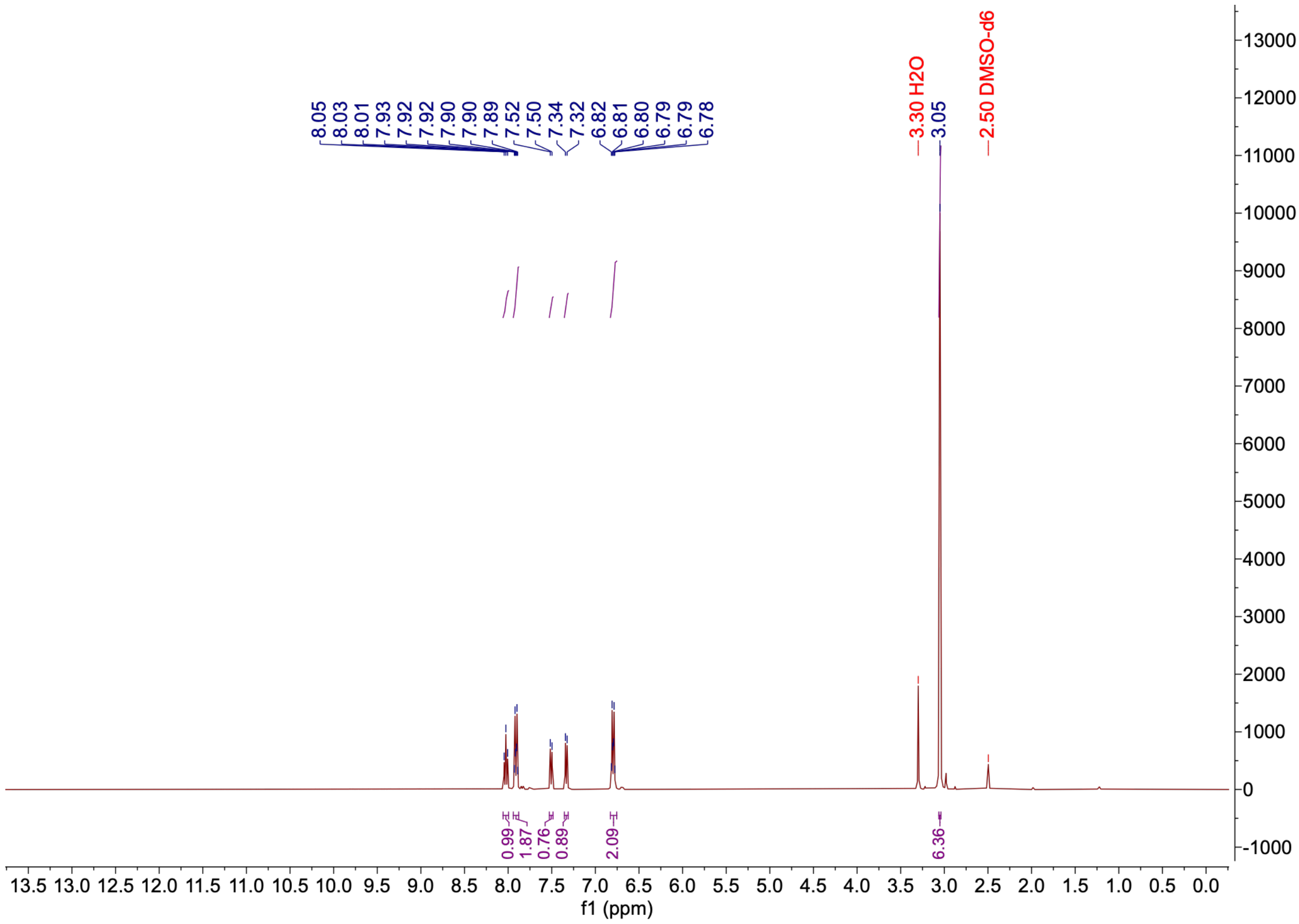

S11: ^{13}CNMR compound **7e** (DMSO-d_6)

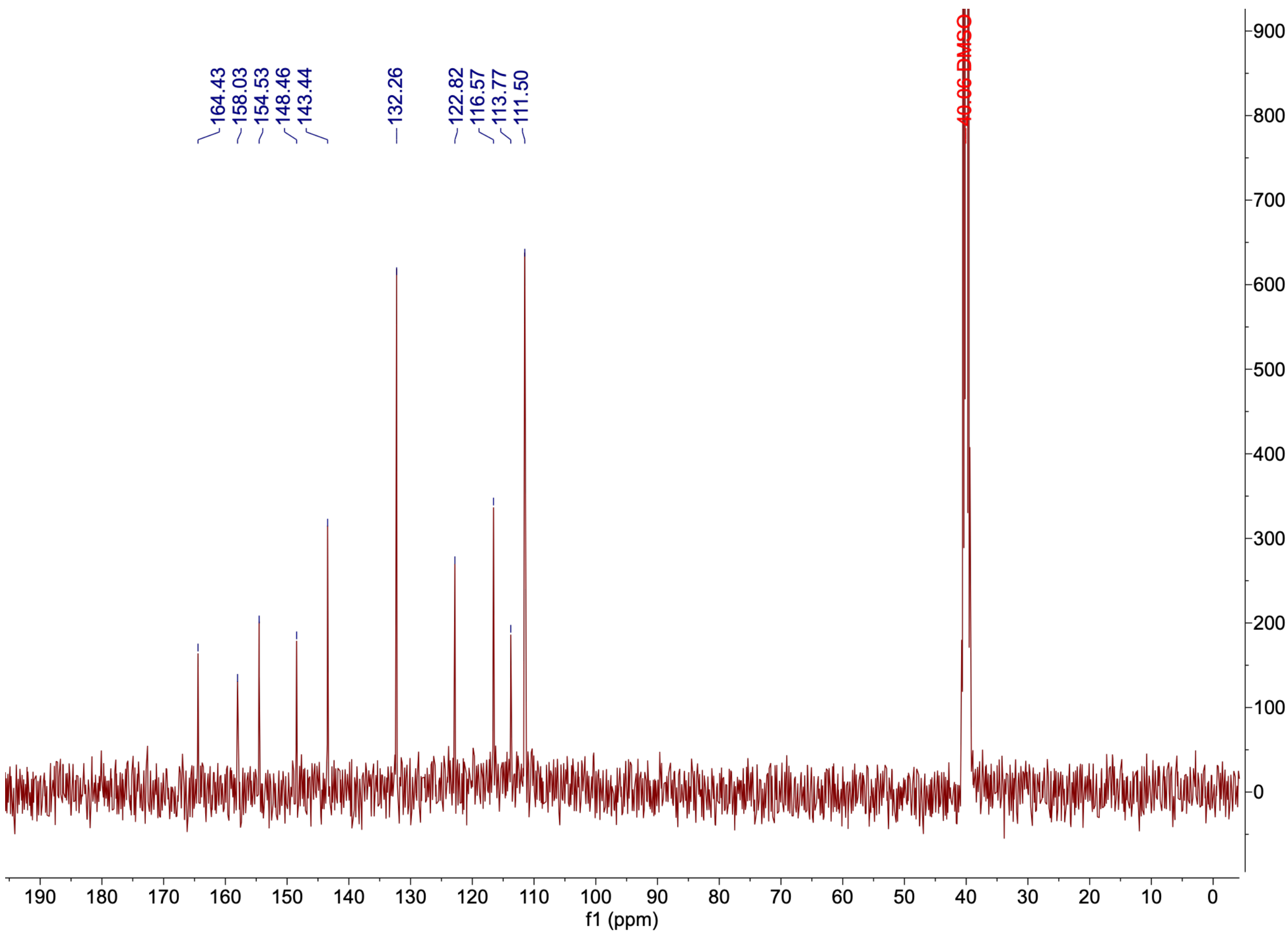

S12: ^{1}HNMR compound **7f** (DMSO-d_6)

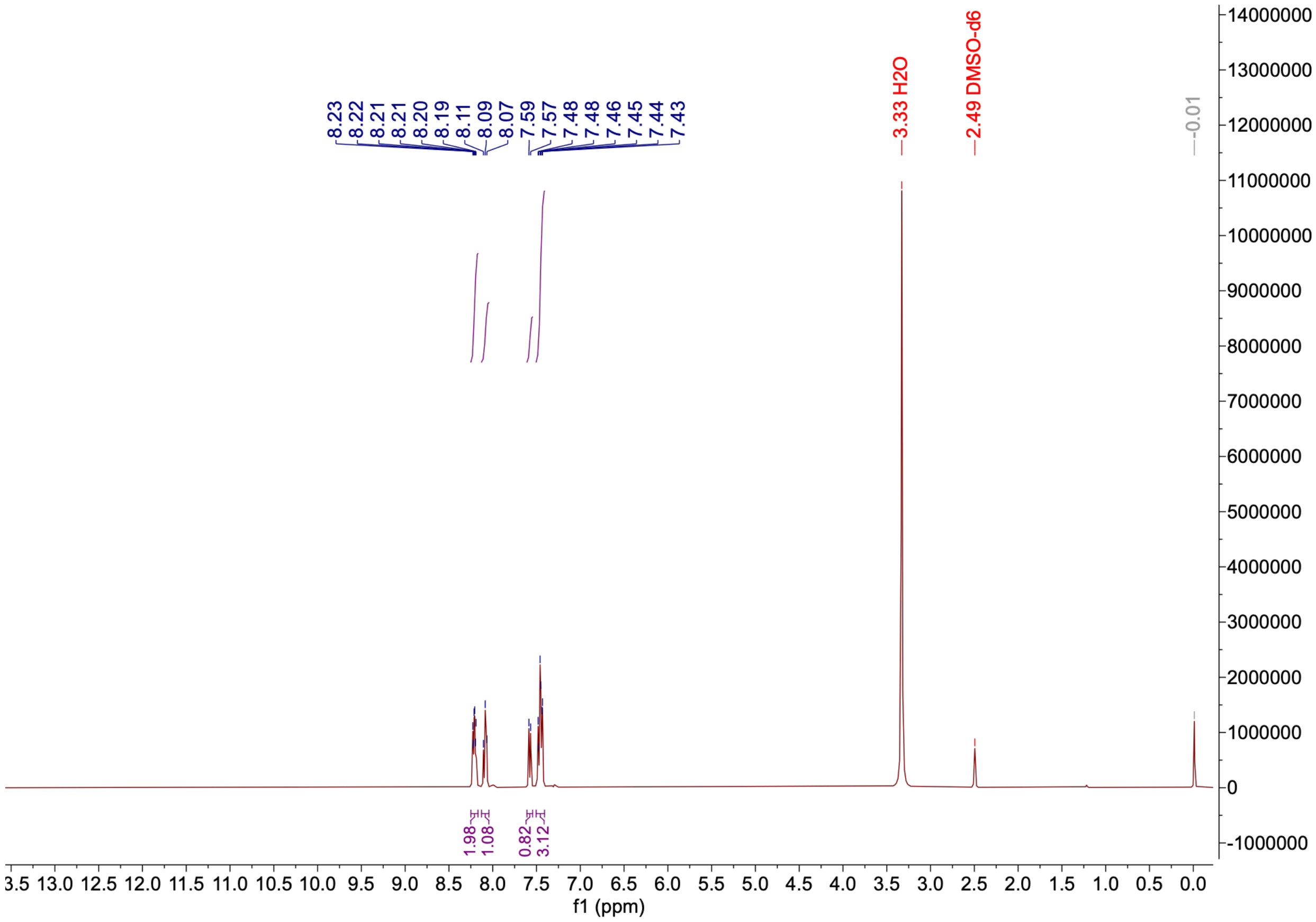

S13: ^{13}CNMR compound **7f** (DMSO-d_6)

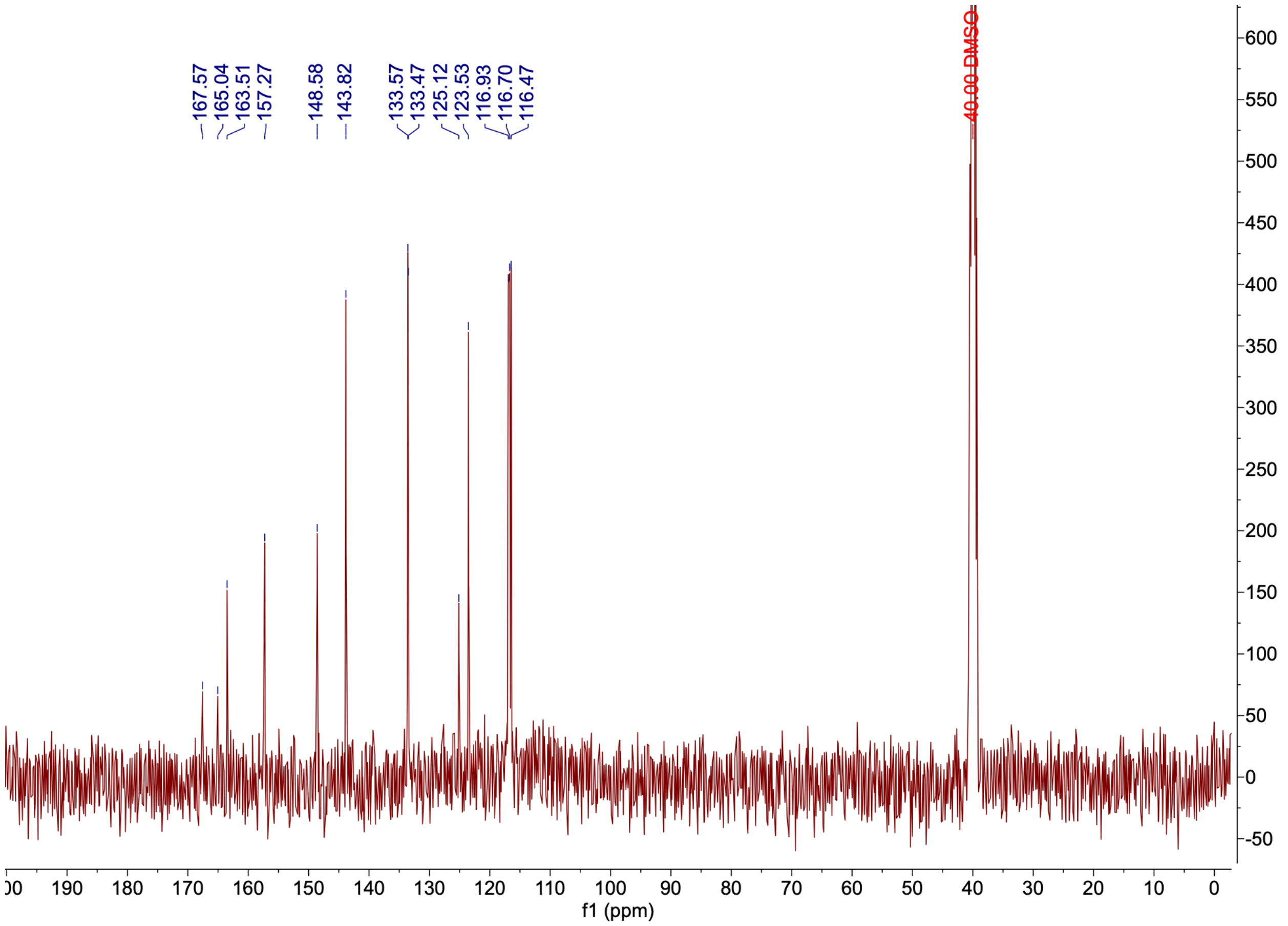

S14: ^{1}HNMR compound **7g** (DMSO-d$_6$)

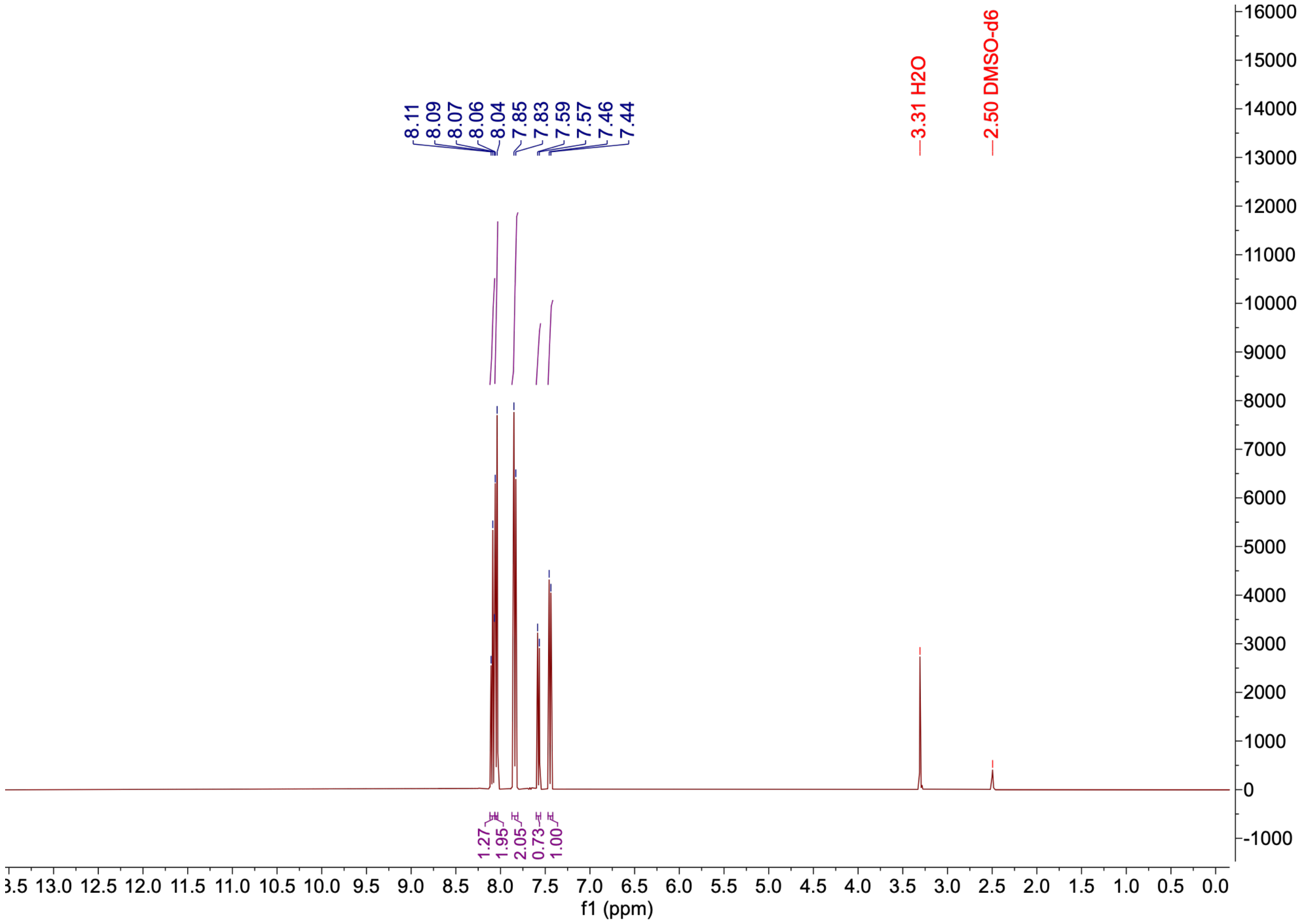

S15: ^{13}CNMR compound **7g** (DMSO-d$_6$)

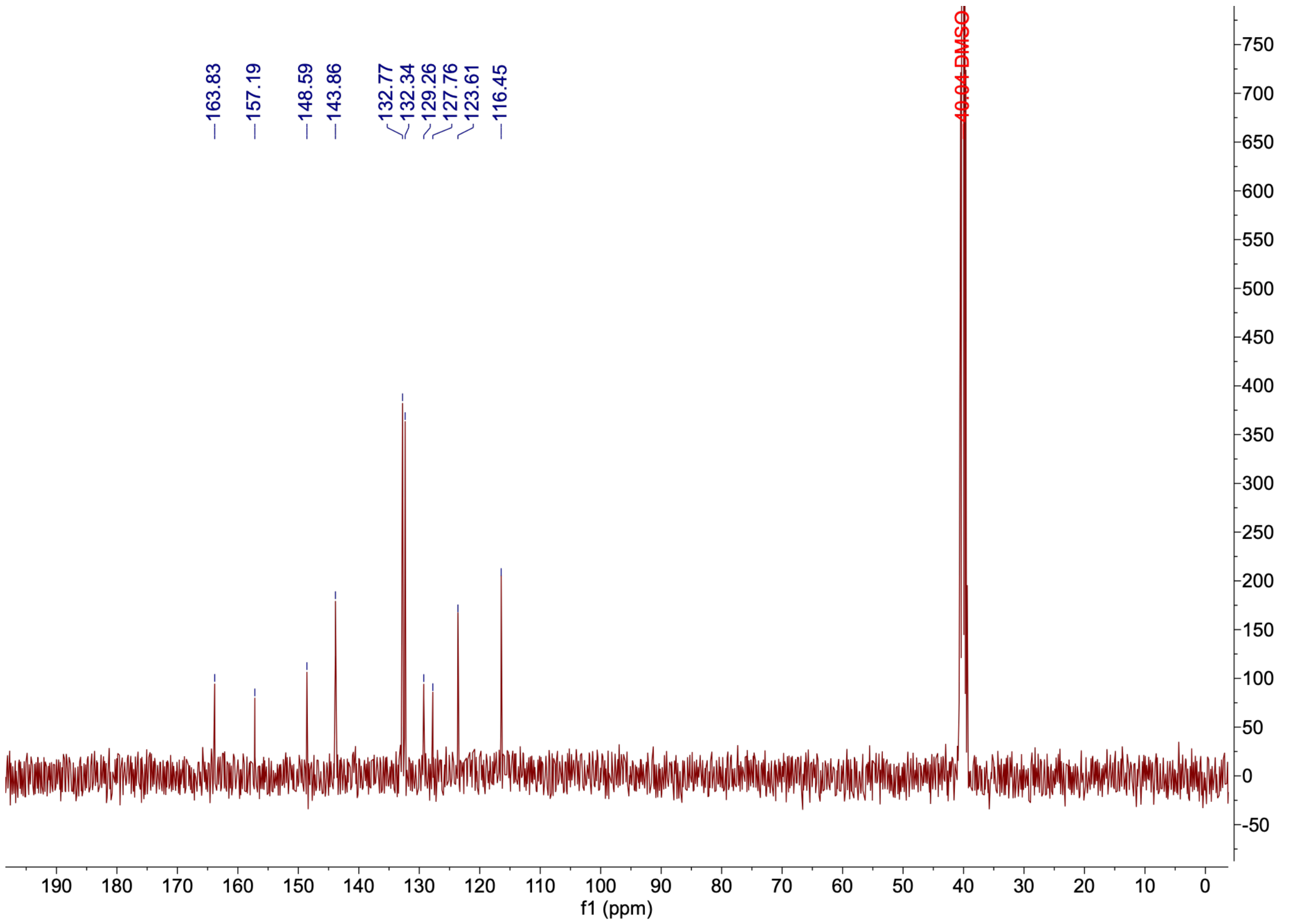

S16: ^{1}HNMR compound **7h** (DMSO-d_6)

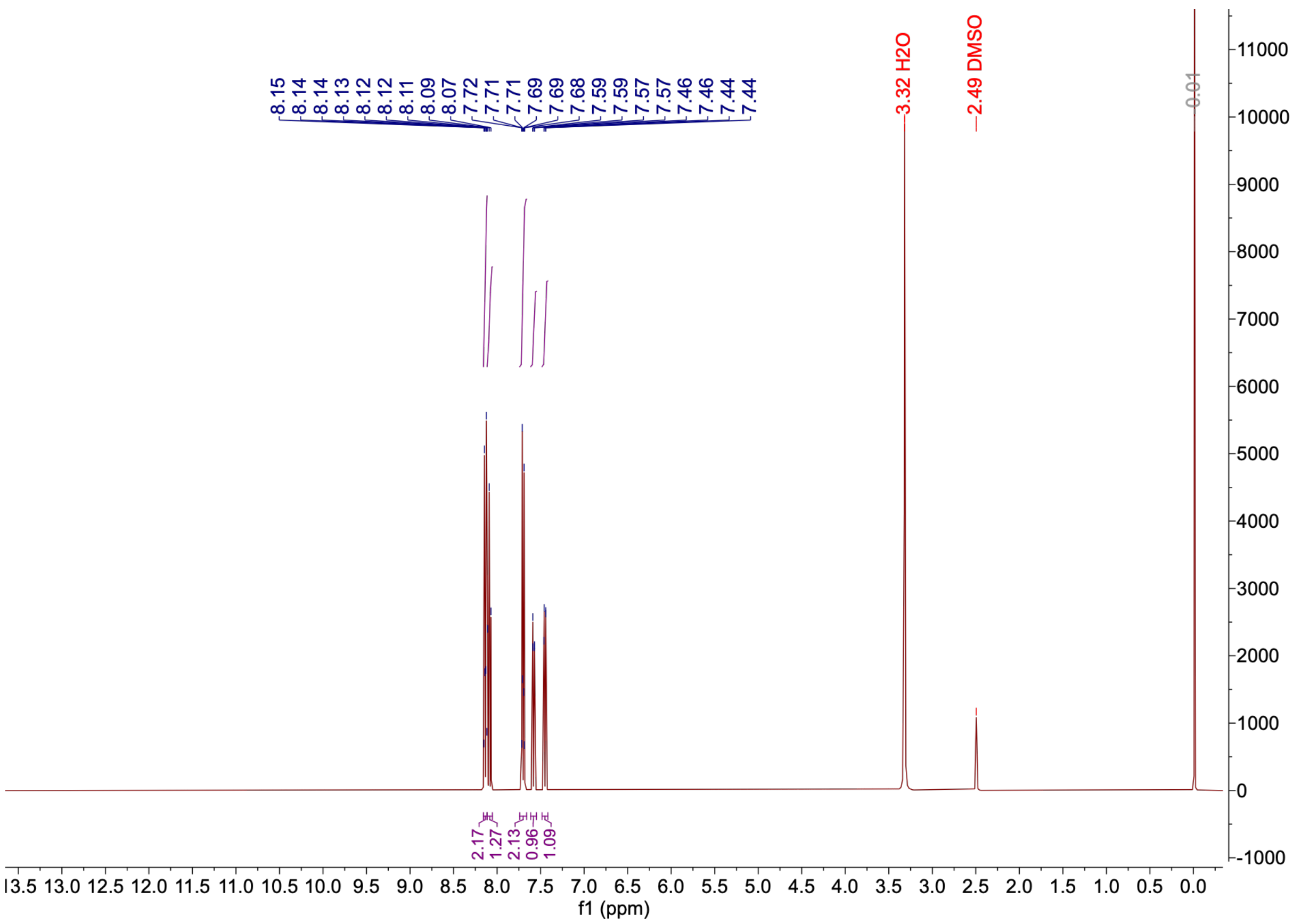

S17: ^{13}CNMR compound **7h** (DMSO-d_6)

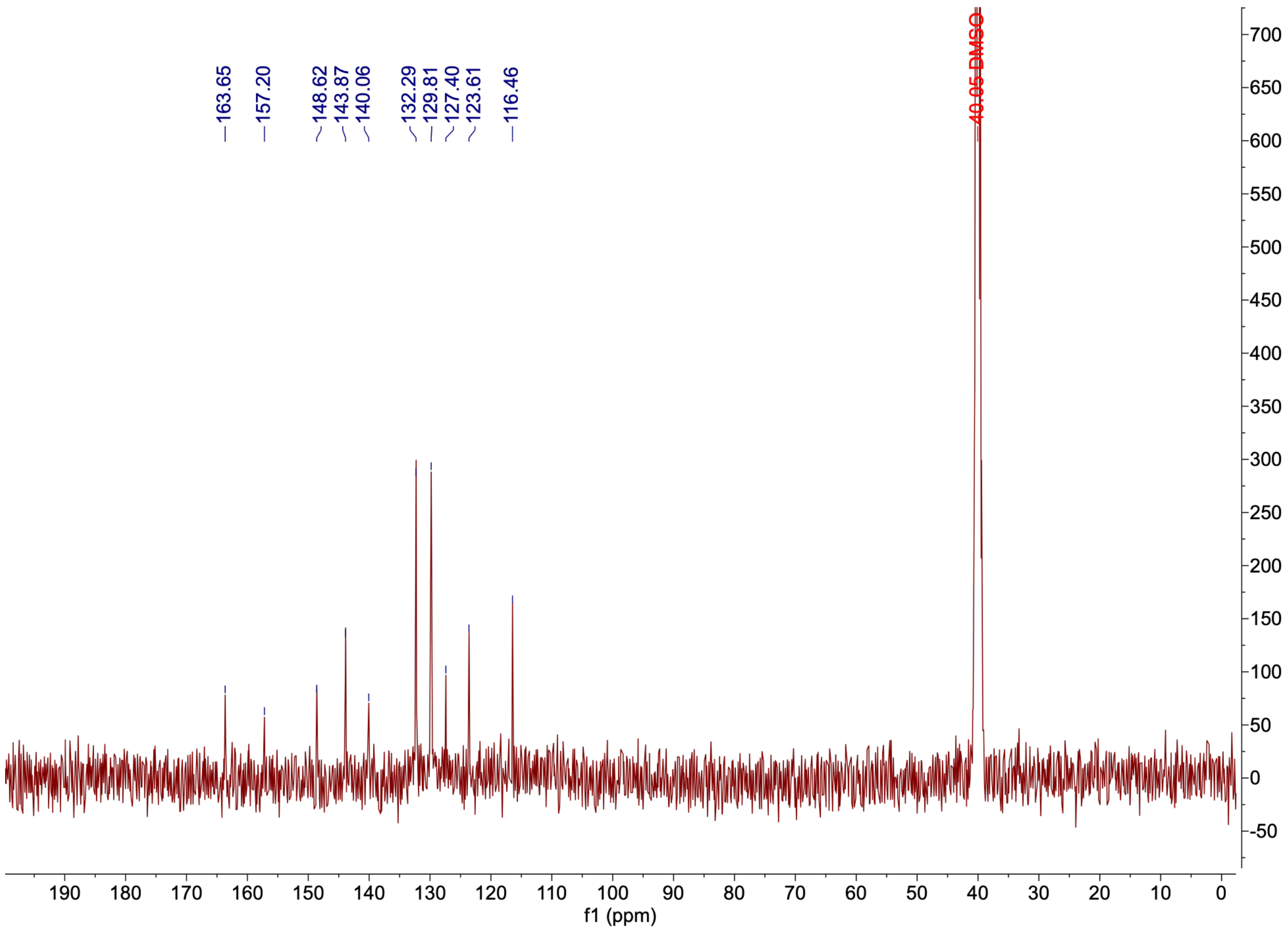

S18: ^{1}HNMR compound **7i** (DMSO-d$_6$)

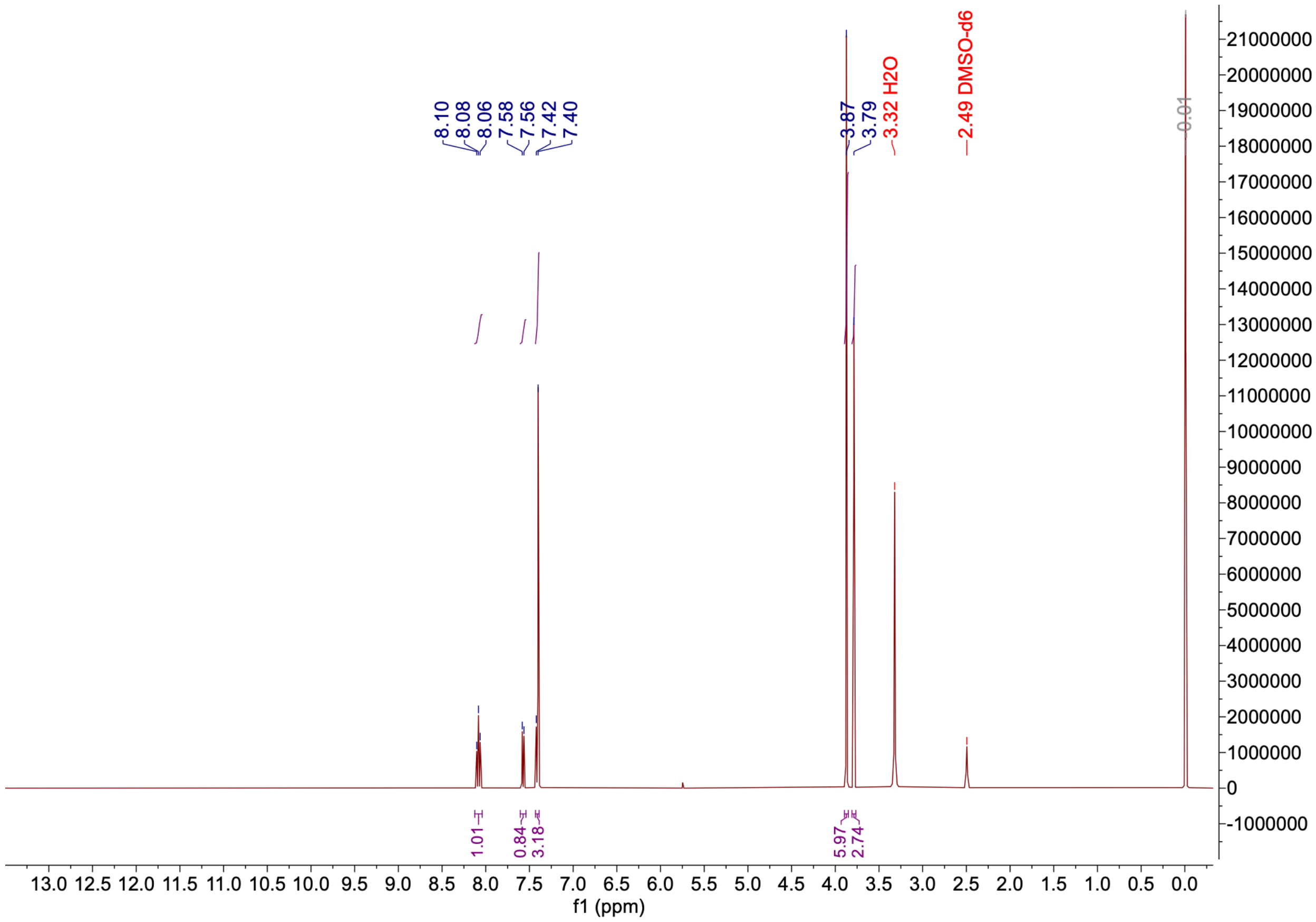

S19: ^{13}CNMR compound **7i** (DMSO-d$_6$)

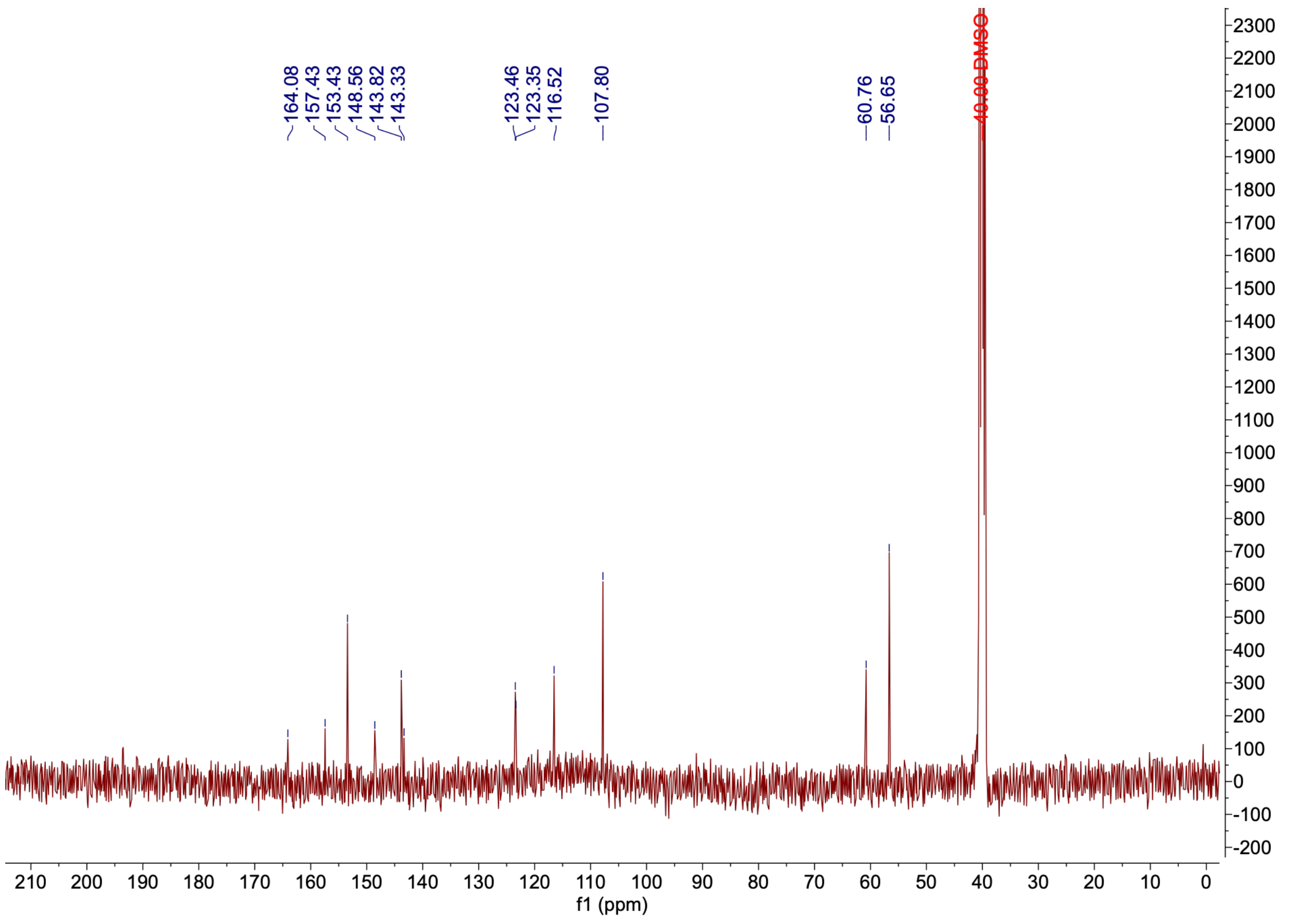

S20: ^{1}HNMR compound **7j** (DMSO-d_6)

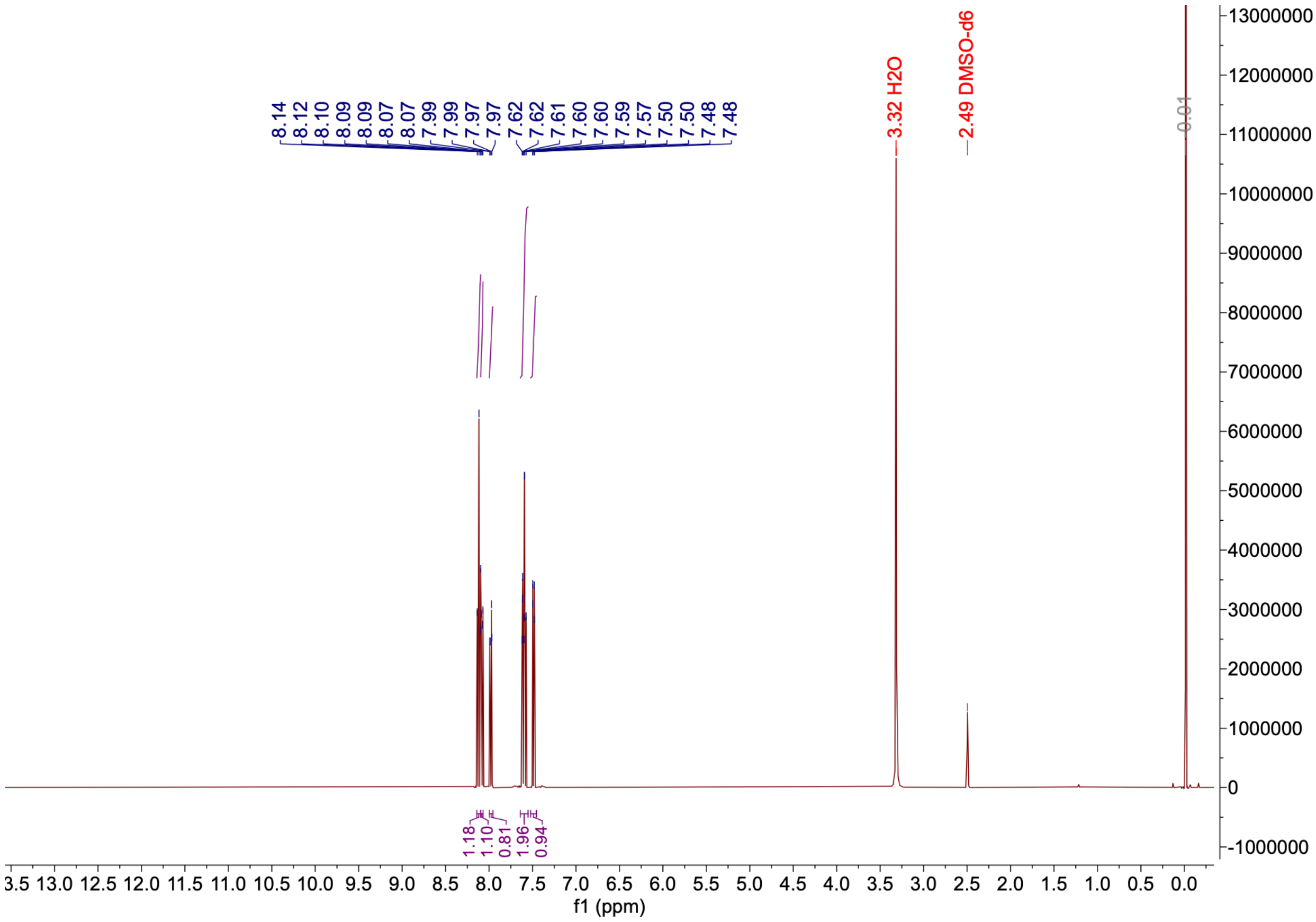

S21: ^{13}CNMR compound **7j** (DMSO-d_6)

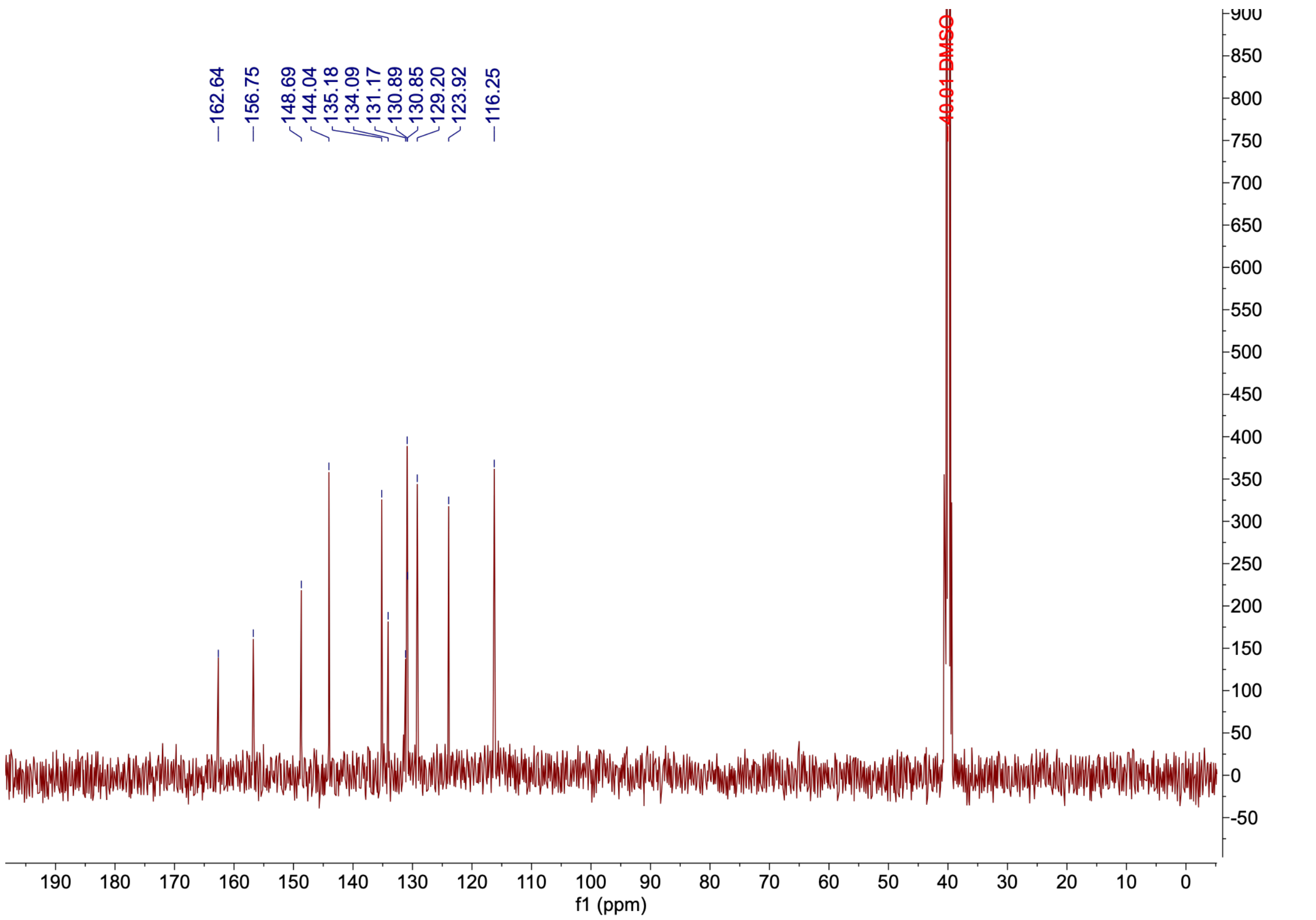

S22: ^{1}HNMR compound **7k** (DMSO-d_6)

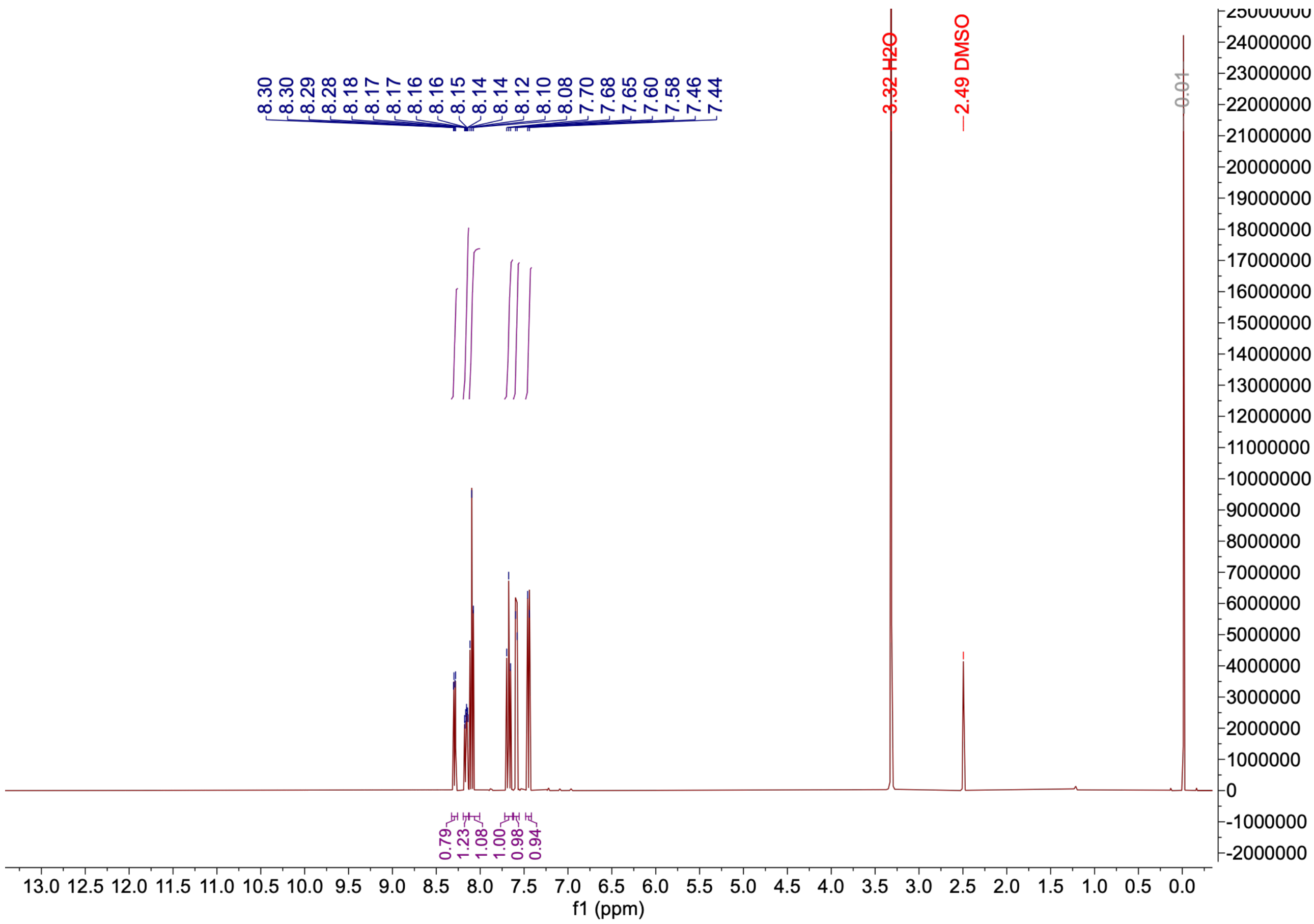

S23: ^{13}CNMR compound **7k** (DMSO-d_6)

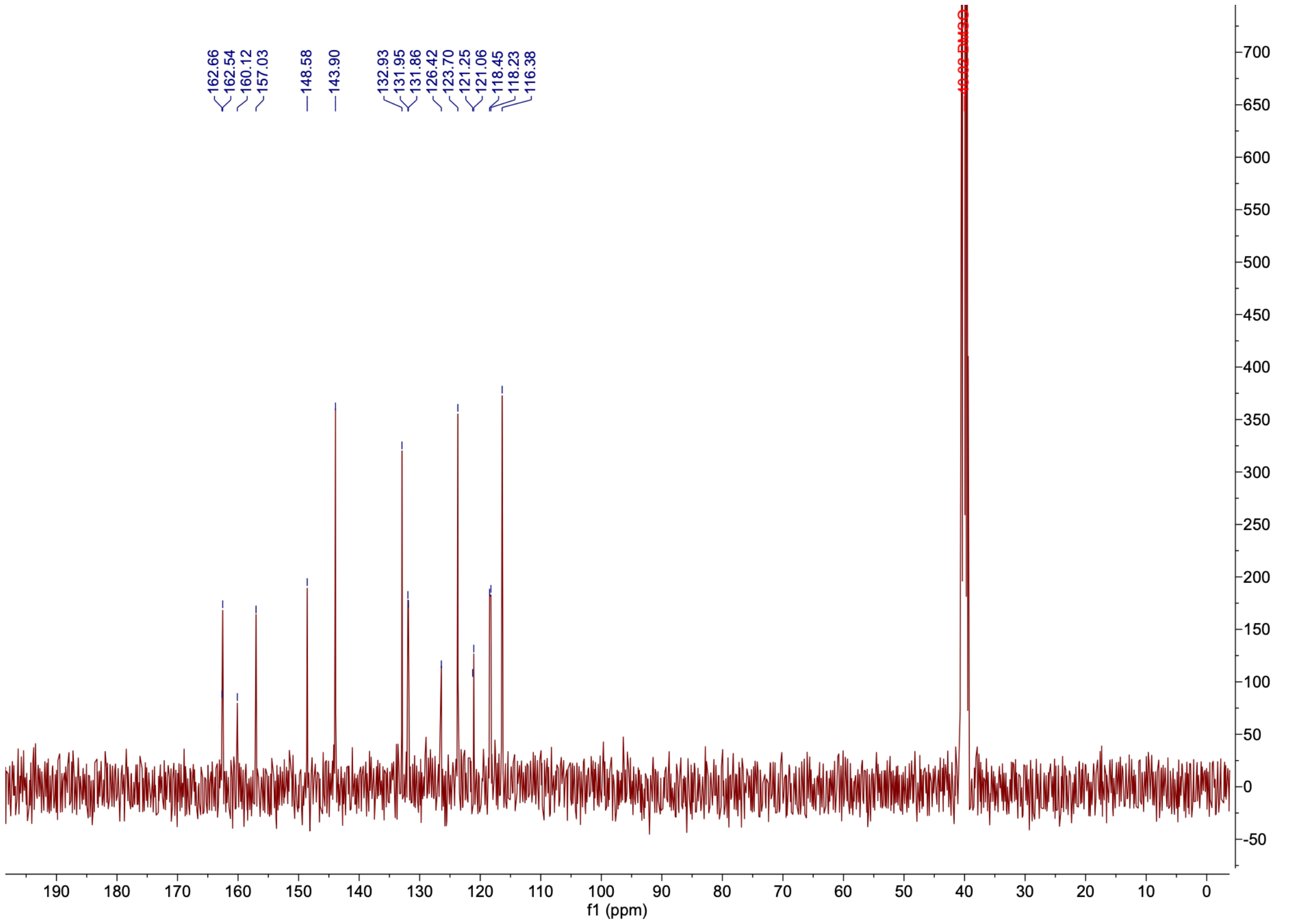

S24: ^{1}HNMR compound **7l** (DMSO-d_6)

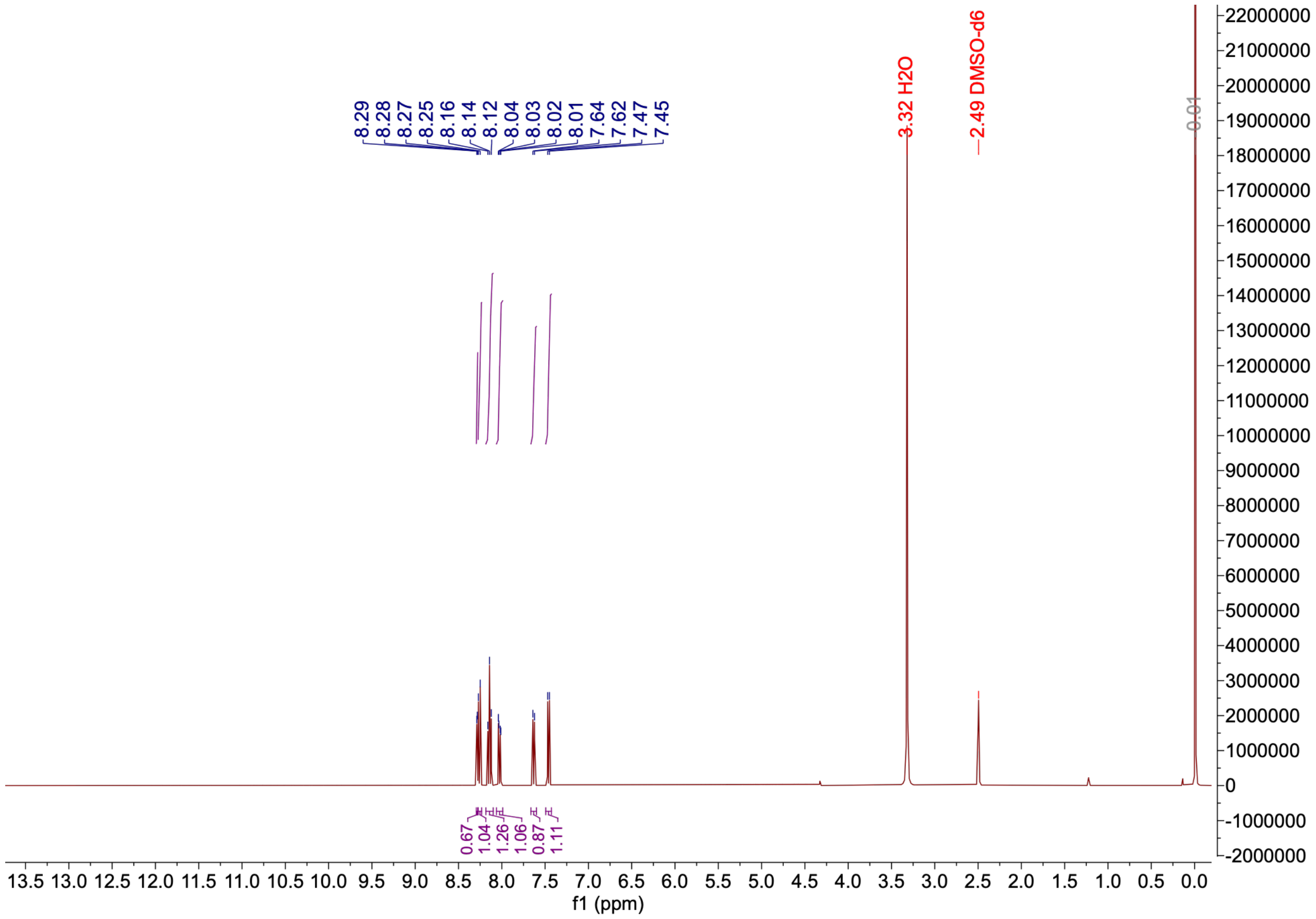

S25: ^{13}CNMR compound **7l** (DMSO-d_6)

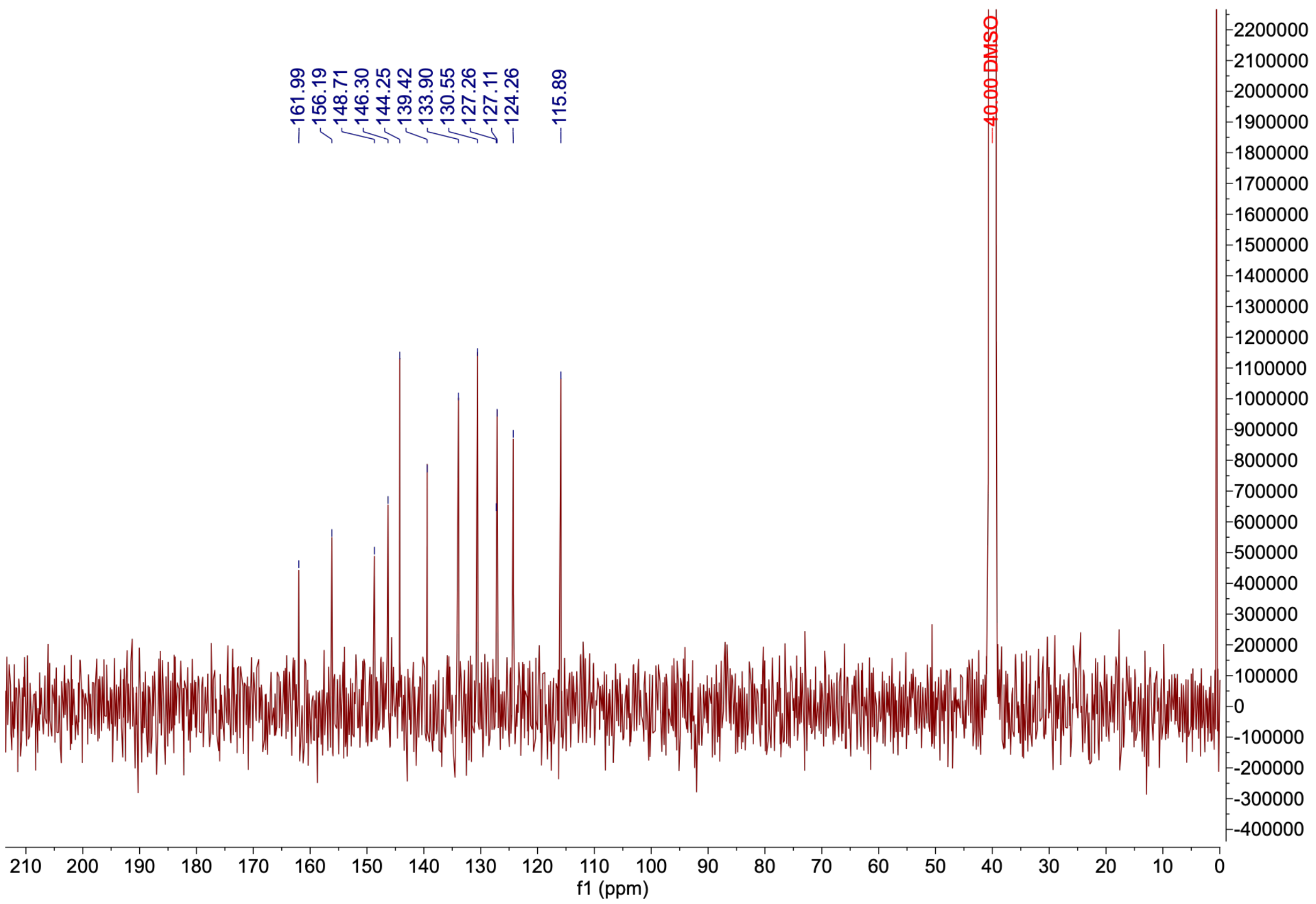

S26: ^{1}HNMR compound **7m** (DMSO-d_6)

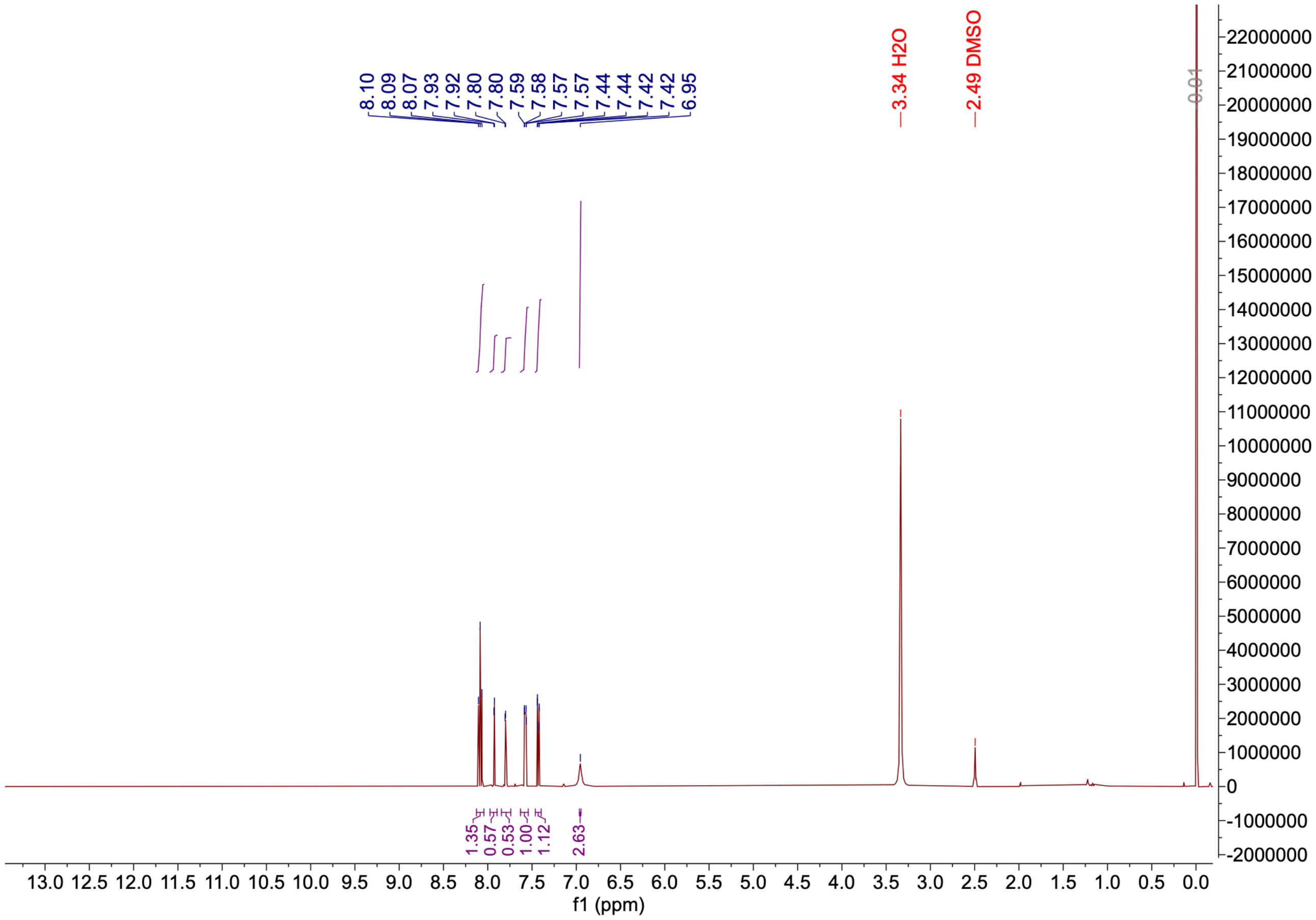

S27: ^{13}CNMR compound **7m** (DMSO-d_6)

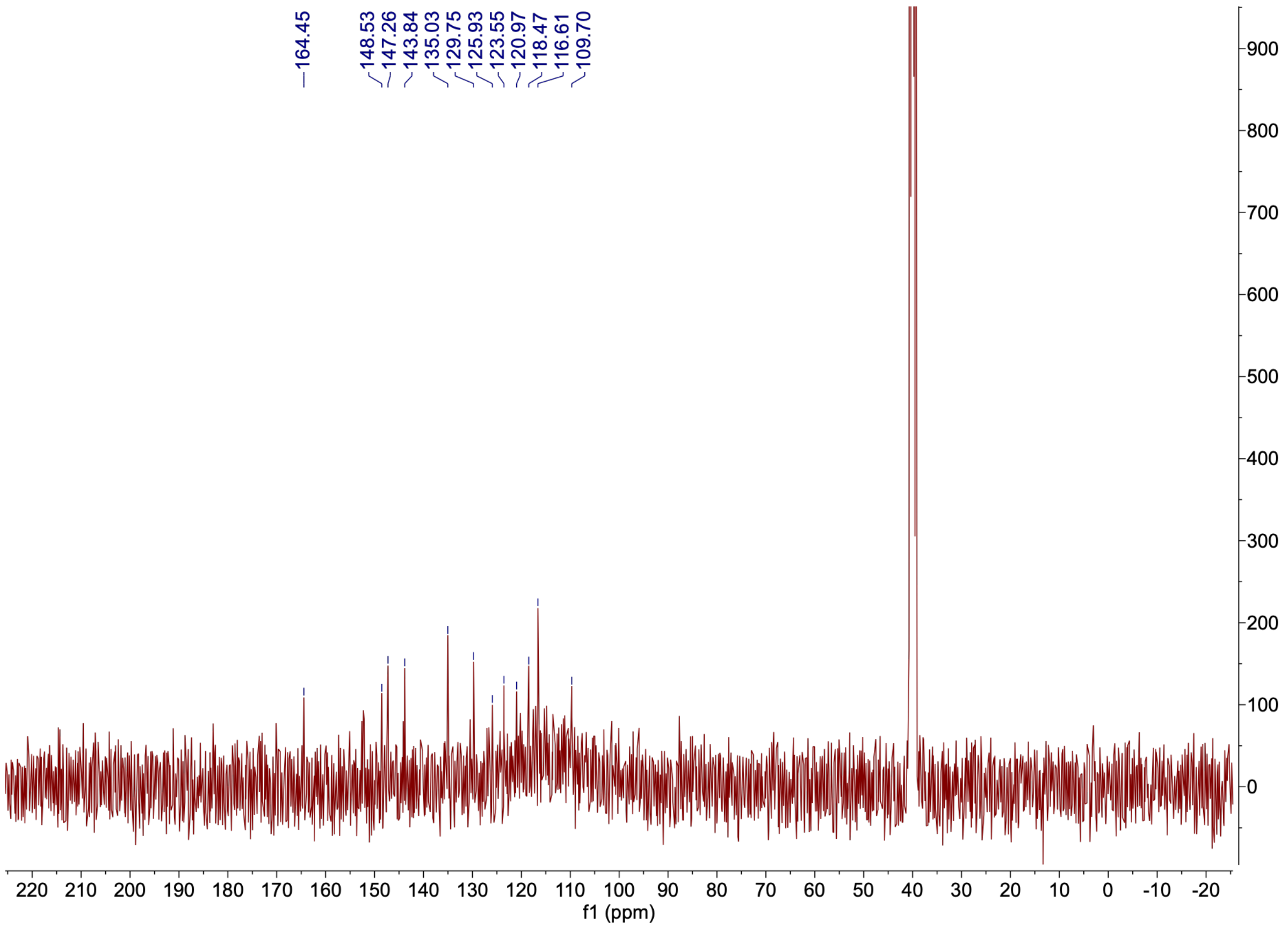

Supplement: Supplementary file 1 [file ao6c06714_si_001.pdf]
